# Antimicrobial Activity of Copper(II), Nickel(II) and Zinc(II) Complexes with Semicarbazone and Thiosemicarbazone Ligands Derived from Substituted Salicylaldehydes

**DOI:** 10.3390/molecules30112329

**Published:** 2025-05-26

**Authors:** Alessio Zavaroni, Luca Rigamonti, Franco Bisceglie, Mauro Carcelli, Giorgio Pelosi, Giovanna Angela Gentilomi, Dominga Rogolino, Francesca Bonvicini

**Affiliations:** 1 Department of Chemistry, Life Sciences and Environmental Sustainability, University of Parma, Parco Area delle Scienze 17/A, 43124 Parma, Italy; alessio.zavaroni@unipr.it (A.Z.); franco.bisceglie@unipr.it (F.B.); mauro.carcelli@unipr.it (M.C.); giorgio.pelosi@unipr.it (G.P.); dominga.rogolino@unipr.it (D.R.); 2 Dipartimento di Scienze Chimiche e Geologiche, Università degli Studi di Modena e Reggio Emilia, Via G. Campi 103, 41125 Modena, Italy; luca.rigamonti@unimore.it; 3 Department of Pharmacy and Biotechnology, University of Bologna, Via Massarenti 9, 40138 Bologna, Italy; giovanna.gentilomi@unibo.it; 4 Microbiology Unit, IRCCS Azienda Ospedaliero-Universitaria di Bologna, Via Massarenti 9, 40138 Bologna, Italy

**Keywords:** metalloantibiotics, acylhydrazone, thiosemicarbazone, antimicrobial activity, cytotoxicity, hemolytic properties, cleavage assay

## Abstract

Antibiotic resistance is a problem repeatedly reported by health authorities. Metalloantibiotics, i.e., biologically active compounds containing one or more metal ions, can be an important resource in the fight against bacteria and fungi. Here, we report the results obtained with a panel of copper(II), nickel(II) and zinc(II) complexes with thiosemicarbazone, semicarbazone and acylhydrazone ligands on *Staphylococcus aureus*, *Escherichia coli* and *Candida albicans*, taken as model systems of human pathogens. To increase the solubility in water, the sulfonic group was introduced on some of the ligands, isolating them as sodium salts (NaH_2_L4-NaH_2_L7). Complexes **1**–**14** were isolated, fully characterized and the X-ray structures of **11**, **12** and **13** were obtained. While all the ligands have no antimicrobial activity, the copper(II) complexes **1** and **4** and the nickel(II) complex **2**, obtained from thiosemicarbazone ligands, showed good activity, in particular against *S. aureus*; these complexes were investigated in depth, calculating their respective IC_50_ values (4.2 μM, 3.5 μM and 61.8 μM, respectively). It should be noted that nickel(II) complex **2** does not show hemolytic activity and has a favorable SI value. While all the copper(II) complexes completely degraded the plasmid DNA in presence of H_2_O_2_, nickel(II) complex **2** cleaved the plasmid DNA leading to the formation of the relaxed nicked conformation, thus suggesting a different mechanism of action.

## 1. Introduction

One of the main goals of bioinorganic chemistry—an interdisciplinary field of scientific research focused on the role of metals in biological systems—is to introduce metal complexes into the repertoire of conventional drugs used in clinical practice. Cisplatin, widely used in cancer treatment, should not remain an isolated case, nor should metal-based drugs be limited to chemotherapy in addressing the growing global concern over both existing and emerging diseases. In recent years, there has been a renewed interest in so-called metal-antibiotics, metal complexes aimed at the treatment of fungi and bacteria that are increasingly resistant to conventional treatments [1,2]. Two particularly significant events that favored and accompanied this renewal of research were the alarm raised by the World Health Organization regarding the spread of antibiotic-resistant bacteria [3] and the initiative coordinated by the group of Angelo Frei at University of Queensland (Australia) and Mark Blaskovich in Bern (Switzerland), where they founded the community for Open Antimicrobial Drug Discovery (CO-ADD) [4]. The antifungal activity of some copper compounds is well known, owing to the introduction of the Bordeaux mixture at the end of the 19th century, as is the strong antibacterial activity of ZnO powder. However, there is a need to identify more effective substances that can achieve good pathogen control but, at the same time, require diminished amounts of metal ions, thereby reducing potential risks for health and the environment. In this regard, metal complexes could represent an important resource, as they combine the biological activity of the metal ion with the ability, by selecting an appropriate ligand, to effectively target pathogens and exert a synergistic effect with the metal ion itself.

**Figure 1:**
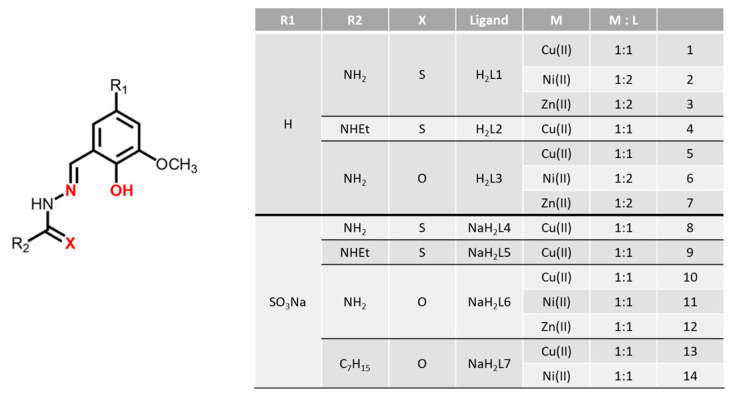
Scheme of the ligands and table with the corresponding metal complexes.

Some encouraging results reported in the scientific literature [5,6,7] have led us to evaluate the possibility of undertaking a screening of the antimicrobial properties of a series of ligands and their related metal complexes that we have been studying in recent years [8,9]. Here, we report on the characterization of some metal complexes with semicarbazone/acylhydrazone and thiosemicarbazone ligands and copper(II), nickel(II) and zinc(II) ions (Figure 1). Thiosemicarbazones were studied for different biological properties [9,10], including for their antibacterial activities [11]. Severe limitations to their practical applications come from their generally poor solubility in water, and the same problem afflicts their metal complexes; an attempt was made to overcome this limitation by introducing a sulfonate group (R = -SO_3_^−^) into the thiosemicarbazone skeleton (Figure 1) [8]. We further expand the panel of ligand and metal complexes in order to investigate the role of the donor atom (sulfur or oxygen) with both sulfonated and non-sulfonated ligands, as well as to study the role of the metal ions (copper, nickel or zinc) in determining antimicrobial activity.

The herein synthetized metal complexes, together with the semicarbazone/acylhydrazone and thiosemicarbazone ligands, were investigated for their in vitro inhibitory activity against *Staphylococcus aureus*, *Escherichia coli* and *Candida albicans*, selected as representative strains for Gram-positive and Gram-negative bacteria and as fungal model system. All the compounds were also evaluated for their overall safety in regard to human cells, as it is well known that metal ions, such as copper(II), nickel(II) and zinc(II) ions, playing a pivotal role in many biological processes, may elicit cellular toxicity [12,13].

## 2. Results and Discussion

### 2.1. Synthesis

**Scheme 1:**
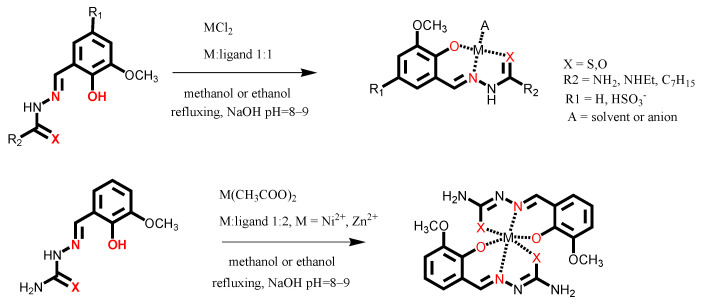
Synthesis of the metal complexes.

The synthesis and characterization of the ligands H_2_L1, H_2_L2, H_2_L3, NaH_2_L4 and NaH_2_L5 were previously described [8,9,10]. The new sulfonated ligands NaH_2_L6 and NaH_2_L7 were obtained according to Scheme S1 [8], and spectroscopic data are reported in Figures S1 and S2. The metal complexes were synthesized according to Scheme 1.

Copper(II) complexes **1** and **4** (Figure 1), previously studied for their anticancer activity [9], present the general formula [Cu(HL)Cl].nH_2_O; the ligand (H_2_L1 or H_2_L2, respectively) is monodeprotonated and *O,N,S* tridentate, with a chlorido anion completing the coordination sphere of the metal ion. The semicarbazonic ligand H_2_L3 shows the same coordinative behavior towards copper(II) ion as its thiosemicarbazonic analogue H_2_L1, giving the complex **5** [Cu(HL3)Cl]. In the IR spectrum of **5**, in fact, a shift of both the C=O and C=N stretching bands can be observed (from 1668 and 1581cm^−1^ in the free ligand to 1654 and 1537 cm^−1^ in the complex, respectively), underlying the involvement of both the carbonyl oxygen and the iminic nitrogen in coordination (Figure S3). Elemental analysis and ESI-MS spectrometry (Figure S4) support the idea that the monodeprotonated ligand is *O,N,O* tridentate and that a chlorine atom completes the coordination of the copper ion. To explore the role of the metal ion on the biological activity of the complexes, and hoping to acquire selectivity towards bacterial strains, we also prepare the nickel(II) and zinc(II) complexes with the ligands H_2_L1 and H_2_L3, isolating compounds **2**, **3**, **6** and **7**, with general formula [M(HL)_2_]·nH_2_O (Figure 1). We employed a 2:1 ligand-to-metal ratio with these ligands, because previous investigations highlighted a similar cytotoxic profile but decreased genotoxicity for 2:1 nickel complexes with respect to the 1:1 ones [14]. In all cases, elemental analyses are in accord with a 2:1 stoichiometry, where the two ligands are monodeprotonated and coordinated to the nickel (**2** and **6**) or zinc metal center (**3** and **7**). Unfortunately, poor solubility prevented the possibility to record the mass spectra of the complexes. The IR spectra, however, show in all cases the absence of the acetate of the starting metal salt; a slight shift of the iminic stretching band with respect to the free ligand (about 5–15 cm^−1^) suggests an involvement of the iminic nitrogen in coordination. Furthermore, in semicarbazonic complexes **6** and **7**, the stretching band of the C=O group is shifted by about 30–40 cm^−1^ with respect to the free ligand, indicating that the carbonyl oxygen is coordinated to the metal (Figures S5–S8). The ^1^H NMR spectrum of **2** recorded in DMSO-*d*_6_ (Figure S9) shows no signs of paramagnetic effects. In solution, the monodeprotonate ligand probably behaves as *NS* bidentate and a square planar nickel(II) complex is formed, in accordance with what is proposed for a similar nickel(II) complex [15,16]. The diamagnetic nature in the solid state is also confirmed by magnetic measurements at room temperature (*χ*_M_*T* = 0.0594 emu K mol^−1^; *χ*_M_ = molar magnetic susceptibility). In the ^1^H NMR spectra of **2** and **7**, registered in DMSO-*d*_6_, it is also possible to observe a set of signals attributable to several species because of a partial displacement of the ligand (Figures S9 and S10). In both cases, no signals attributable to the acetate ion of the starting metal salt were present. Solubility issues prevented registration of the ^1^H NMR spectra of **3** and **6**.

**Figure 2:**
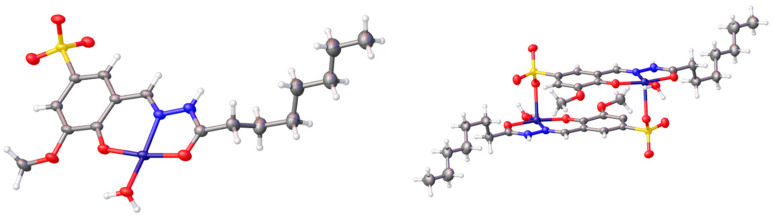
Crystal structure of the molecular unit (**left**) and of the head-to-tail dimers [Cu(HL7)(H_2_O)]_2_ (**right**).

The metal complexes of the sulfonated ligands NaH_2_L6 (Cu(II) **10**; Ni(II) **11**; Zn(II) **12**) and NaH_2_L7 (Cu(II) **13**; Ni(II) **14**) were also synthesized. For the copper complexes **10** and **13** and the nickel complex **14**, the formula [NaM(HL)Cl] nH_2_O is proposed, in accordance with analytical data and what was previously found with the similar compounds **8** and **9** [8]. In the IR spectra of the complexes, the ν(O-H) band around 3460 cm^−1^ disappears because of the deprotonation of the OH group and there is a shift of 15–60 cm^−1^ for the ν(C=N) and ν(C=O) bands (Figures S11, S14 and S15): it is reasonable to propose an *O, N, O* coordination of the ligand as it is confirmed by the obtained X-ray structures. In the ESI-MS spectra of the complexes **10** and **13** in methanol, there are the signals for the species [CuL] and for the dimeric ones (Figures S16 and S17). Effectively, we have previously observed that the metal complexes of the sulfonated ligands tend to rearrange in solution [8]. By slow evaporation of a methanol solution of **13,** crystals of [Cu(HL7)(H_2_O)]_2_ were obtained, with elimination of NaCl. SC-X-ray diffraction analysis revealed that the complex forms head-to-tail dimers in the solid state (Figure 2). The copper(II) ion has a square planar coordination with the *O, N, O* tridentate ligand and a water molecule; an additional apical interaction with the negatively charged oxygen of the sulfonate group from a neighboring molecule completes the [Cu(HL7)(H_2_O)]_2_ dimer. This arrangement has been previously observed in zwitterionic copper complexes containing sulfonate moieties [16,17,18,19] and in a related thiosemicarbazone analogue [8]. It is reasonable to propose that, during crystal formation, once one molecule binds to the oppositely charged fragment of an adjacent molecule, the likelihood of the other end finding a similarly oppositely charged counterpart within the same molecule is quite high.

The tendency of the metal complexes of the sulfonated ligands to rearrange themselves by eliminating NaCl and eventually dimerize is corroborated by the SC-X-ray diffraction analyses of the crystals obtained by recrystallization of **11**, i.e., [Ni(HL6)(H_2_O)_2_]_2_ [Ni(HL6)(H_2_O)_3_], and **12,** i.e., [Zn(HL6)(H_2_O)_2_].

**Figure 3:**
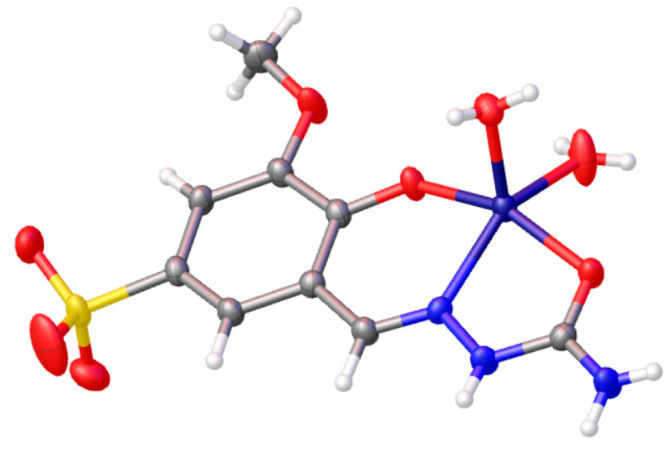
Crystal structure of the zinc(II) complex [Zn(HL6)(H_2_O)_2_].

In the crystal structure of [Zn(HL6)(H_2_O)_2_], the *d*^10^ metal ion presents a slightly distorted square pyramidal coordination that includes in the basal plane one water molecule together with the three donor atoms of the ligand and a further water molecule occupying the apical position (Figure 3). Another free crystallization water molecule is found in the crystal and plays a role in the packing, forming four hydrogen bonds connecting other subunits: two of them as donors with sulfonyl groups of two adjacent molecules, one as a receiver from the hydrogen of a terminal NH_2_ amino group and the fourth from a protonated hydrazide nitrogen of another molecule.

**Figure 4:**
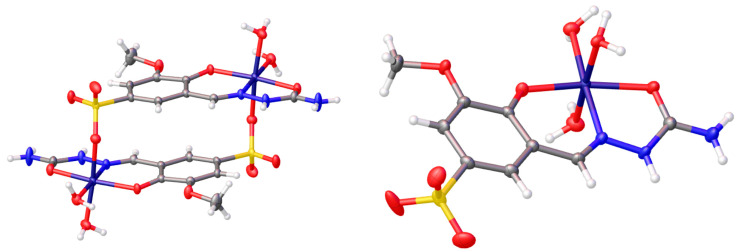
Crystal structure of the two differently coordinated nickel(II) ions in [Ni(HL6)(H_2_O)_2_]_2_ [Ni(HL6)(H_2_O)_3_].

The crystal structure of the nickel complex [Ni(HL6)(H_2_O)_2_]_2_[Ni(HL6)(H_2_O)_3_] is instead formed by two differently coordinated Ni ions in the asymmetric unit (Figure 4). One has a nickel ion that coordinates in an octahedral fashion six donor atoms, three from the ligand and the remaining three occupied by water molecules. In the second molecule, one of the apical water molecules is occupied by the oxygen of a sulfonyl group of an adjacent analogue molecule with which it forms a head to tail dimer, as already observed in [Cu(HL7)(H_2_O)]_2_. In both cases, the double positive charge of the metal ion is balanced by the two negative charges of the deprotonated hydroxyl group of the aromatic ring and by the charge of the sulfonyl group. In the crystal packing, an additional water molecule connects the complexes to create a complex network of hydrogen bonds.

### 2.2. Biological Evaluations

The biological properties of the metal complexes were evaluated in vitro against three laboratory strains, *Staphylococcus aureus* ATCC 25923, *Escherichia coli* ATCC 25922, and *Candida albicans* ATCC 10231. The biological studies also included an assessment of the cytotoxicity towards human fibroblasts (HEL 299 CCL-137) and the measurement of the hemolytic activity on human red blood cells (hRBCs), thus obtaining a complete overview on the antimicrobial effectiveness and safety of the metal complexes.

Before carrying out the biological tests, the stability in solution of the newly synthesized sulfonated complexes was evaluated over 72 h by recording their UV spectra (C ≈ 0.25 μM) in 25 mM of HEPES buffer solutions at pH = 7.4 in H_2_O/NaCl 0.9%. No visible variations can be observed in the spectra over 72 h, confirming the stability of the coordination compounds in these conditions. As an example, the UV-visible spectra for compound **13** are reported in the supporting information paragraph (Figure S24). All the tested compounds are soluble in 100% DMSO at 20 mM (stock concentrations) and in the cell culture media at the dilutions used in the biological assays.

#### 2.2.1. In Vitro Antimicrobial Activity

The antimicrobial activity of the metal complexes and of the ligands was assayed using a standardized microdilution method in compliance with the Clinical and Laboratory Standard Institute (CLSI) guidelines; the inhibitory effect of the tested compounds at 100 μM is reported in Table 1 and Table S1.

Some general remarks can be drawn from data reported in Table 1: none of the metal complexes displayed a broad antimicrobial activity against the three tested pathogens, and *S. aureus*, the model system for Gram-positive strains, proved to be the most susceptible species. Indeed, compounds **1**, **2** and **4** completely inhibited *S. aureus* proliferation at 100 μM, while compounds **1** and **4** slightly interfered with fungal proliferation. *E. coli* growth was only marginally reduced (44.2%) by compound **5** compared to the untreated cells. This different susceptibility to metal complexes **1**, **2** and **4** can be ascribed to their different interaction with the multi-layered cellular structures and uptake throughout the cell walls of Gram-positive, Gram-negative and fungal cells. Specifically, the outer membrane of Gram-negative strains and the fungal cell wall create a permeability barrier to molecules, thus reducing the effectiveness of biologically active compounds [20,21,22], including clinically used antibacterial and antifungal drugs [23].

In addition, as the ligands lacked antimicrobial activity at the highest tested concentration (Table S1), the inhibitory potential can be attributed to the complexes and their efficacy in reducing microbial proliferation to the metal ion as well as the related ligands. Indeed, copper(II) complexes displayed a potent inhibitory activity against *S. aureus* and a moderate activity against *C. albicans* when associated with the thiosemicarbazone ligands (H_2_L1 and H_2_L2), while they proved to be ineffective with all the other ligands, thus highlighting the importance of the ligand in the complex. This finding is even more evident when comparing the results obtained in *C. albicans*, in which the percentage values of the fungal growth decreased from 43.6% to 15.6% in relation to ligand lipophilicity in the copper(II) complexes. The enhanced lipophilicity of compound **4** can influence the cellular uptake and accumulation of the metallodrug, thus increasing its activity. As for the nickel(II) series, only the metal complex with H_2_L1 (**2**) proved to selectively inhibit *S. aureus*, confirming a pivotal role for the ligand in directing the effectiveness of the metal ion.

The complexes bearing the sulfonate group (**8**–**14**) are inactive against the tested pathogens, and these results suggest that, while improving water solubility, the introduction of the sulfonate group at the same time reduce lipophilicity, impairing cellular uptake and leading to poor biological activity.

#### 2.2.2. Cytotoxicity and Hemolytic Activity

The metal complexes and the semicarbazone/acylhydrazone and thiosemicarbazone ligands were assayed in vitro on HEL 299 fibroblasts and hRBCs to determine their overall safety in terms of cell viability and hemolytic properties, respectively. Indeed, it is generally recognized that toxicity represents a limiting parameter in the drug discovery pipeline, thus deserving a comprehensive investigation.

Data are reported in Table 2. The metal complexes, regardless of the ion and the ligand, proved to affect HEL 299 viability to different extents, but a general trend is outlined. Indeed, metal complexes with ligands without the sulfonate group (**1**–**7**) displayed an overall higher cytotoxicity compared to the others (**8**–**14**), with copper(II) complexes being the most toxic in the series.

**Figure 5:**
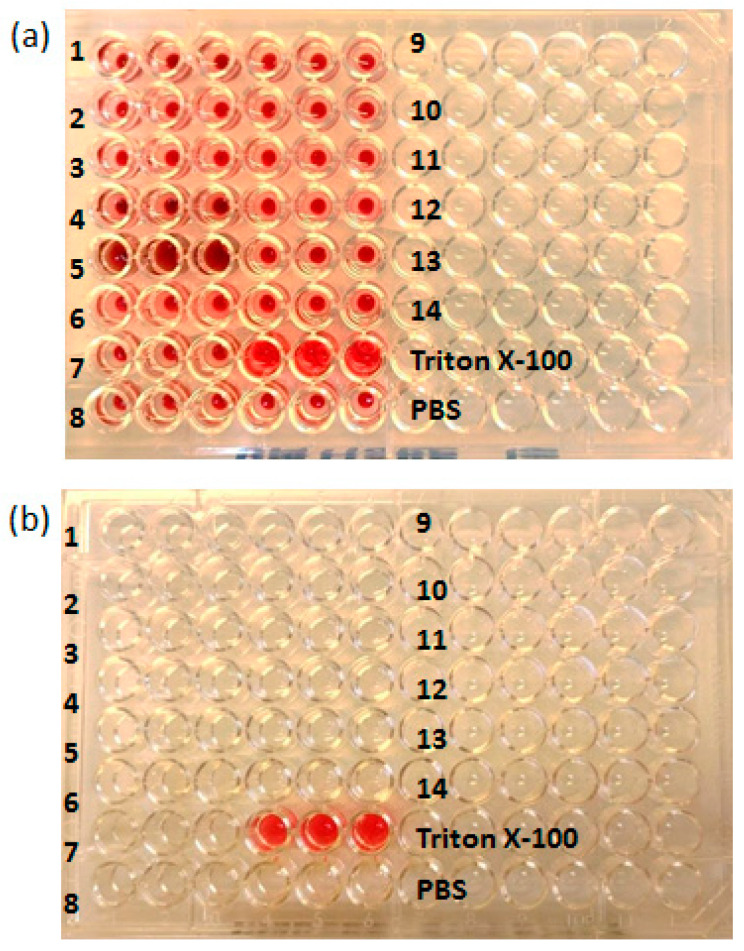
Hemolytic activity of the metal complexes. Representative image for hRBC hemolysis in response to 100 μM of compounds (**a**) and the corresponding supernatants collected for _OD492 nm_ measurements of the hemoglobin released from hRBCs (**b**). Triton X-100 and PBS are used as positive and negative controls, respectively.

As for the hemolytic properties, all metal complexes displayed a negligible effect on hRBCs (Table 2, Figure 5). Overall, these findings indicate that the observed cytotoxicity is related to a block in cell replication rather than to the disruption of cytoplasmic membranes, in agreement with the anti-proliferative potential of some previously described metal complexes (**1** and **4**).

### 2.3. In Vitro DNA Cleavage Assay

The nuclease activity of the metal complexes was investigated by means of gel electrophoresis after incubating the reaction mixtures (100 µM of sample and 200 ng of plasmid DNA) at 37 °C for 2 h. These experimental conditions were conceived in a set of preliminary in vitro studies by using cisplatin as reference metal-drug and hydrogen peroxide to increase the concentration of reactive oxygen species (Figure S20). Thus, the assays with the metal complexes were performed in the absence and presence of H_2_O_2_ at 10 mM to check whether the DNA cleavage and damage take place through a hydrolytic or an oxidative mechanism.

**Figure 6:**
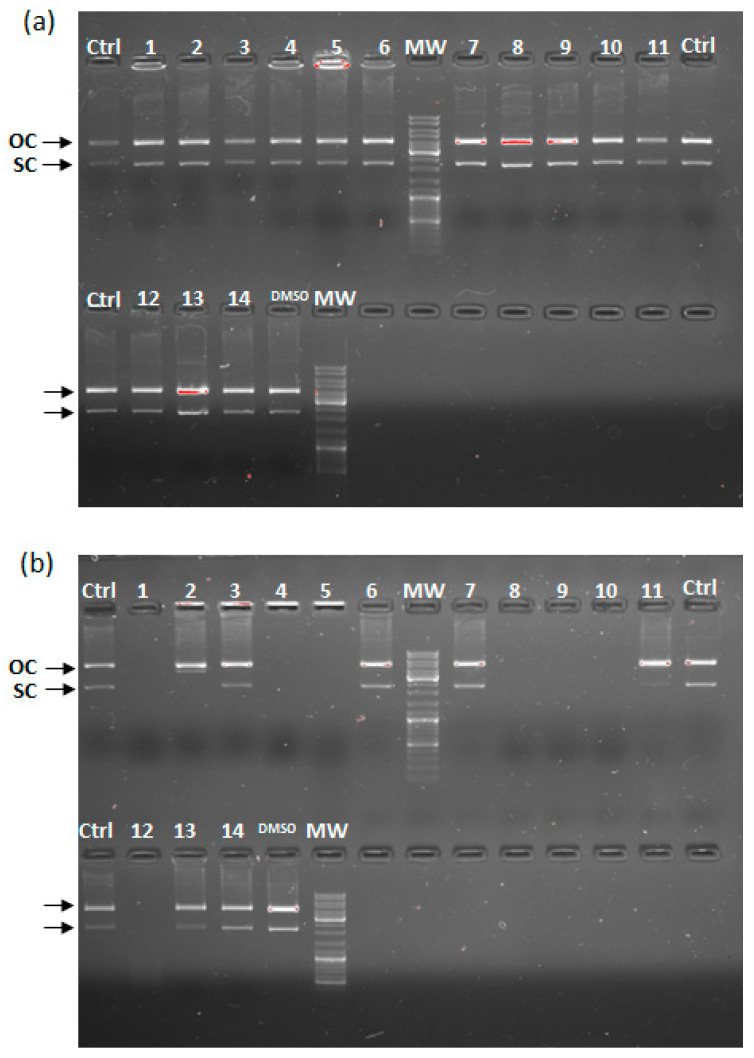
Cleavage experiments of pmaxGFP (3527 bp) in absence (**a**) and presence (**b**) of H_2_O_2_. MW (Gene Ruler DNA ladder mix); Ctrl (pmaxGFP) appears in two forms, the open circular form (OC) and the supercoiled form (SC). The incubation with the metal complexes in the presence of H_2_O_2_ strongly affects the band pattern after the electrophoresis run.

Figure 6 shows that metal complexes in absence of H_2_O_2_ did not interfere with the electrophoretic mobility of the plasmid DNA nor behave as cleaving agents. Indeed, no differences were observed in the pattern of bands between the DNA control and the treated samples, both in the open circular and the supercoiled DNA forms. On the other hand, cleavage experiments in the presence of H_2_O_2_ displayed different results depending on the tested metal complexes, thus indicating that H_2_O_2_ plays a crucial role in the reaction.

Considering the metal complexes with thiosemicarbazone ligands, three different results are depicted as a function of the metal ion; indeed, copper(II) complexes **1** and **4** produced extensive damage to the plasmid DNA, as indicated by the complete disappearance of the bands, while nickel(II) complex **2** promoted the cleavage of only one strand of the plasmid DNA, leading to the formation of the relaxed nicked conformation, i.e., the open circular form. Finally, the zinc(II) complexes were not able to interact with DNA.

Notably, all copper(II) complexes, regardless of the ligands, extensively damaged the DNA, with the only exception being complex **13** with NaH_2_L7, thus indicating the cleaving activity of this metal ion.

### 2.4. Cell-Selective Activity

To shed light on the selective activity of metal complexes **1**, **2** and **4**, the best performing compounds inhibiting microbial proliferation but interfering with HEL 299 metabolism, the IC_50_ and CC_50_ values were measured in dose–response experiments. As reported in Table 3, only the nickel(II) complex (**2**) with the thiosemicarbazone ligand displayed a favorable SI value, indicating a prevalent inhibitory activity against bacterial proliferation rather than a toxic activity on human fibroblasts.

## 3. Materials and Methods

### 3.1. Synthesis

Reagents and solvents were purchased from commercial sources (Sigma-Aldrich, St. Louis, MO, USA). The purity of the new compounds (≥95%) was determined by elemental analysis. Elemental analyses were performed by using a CHNS FlashSmart Thermo Fisher analyzer (Thermo Fisher Scientific, Waltham, MA, USA). ^1^H-NMR (400 MHz) and ^13^C-NMR (125 MHz) spectra were recorded at 25 °C on a Bruker Avance 400 FT spectrophotometer (Bruker, Billerica, MA, USA). The ATR-IR spectra (4000–400 cm^−1^) were recorded by means of a Perkin-Elmer spectrophotometer (PerkinElmer, Waltham, MA, USA) by using a diamond crystal plate. Electrospray mass spectral analyses (ESI-MS) were obtained with an electrospray ionization (ESI) time-of-flight Micromass 4LCZ spectrometer (Waters Micromass, Milford, MA, USA); samples were previously dissolved in methanol. Magnetic measurements at room temperature were performed with a magnetic susceptibility balance, Sherwood Scientific Ltd. (Cambridge, UK). The UV–vis spectra were collected using a Thermo Evolution 260 Bio spectrophotometer provided with a thermo-statting Peltier device and quartz cuvettes with 1 cm path length (Thermo Fisher Scientific, Waltham, MA, USA).

Single-crystal X-ray diffraction analyses were carried out with a Bruker D8 Venture diffractometer equipped with a kappa goniometer and an Oxford cryosystem. The main X-ray crystallographic data are reported in the Supporting Information. Lorentz polarization and absorption correction were applied (SADABS procedure, [24]). The phase problem was solved by direct methods and the structures were refined by full-matrix least-squares on all F2 using SHELXL [25,26] (OLEX2 suite of programs [27]). The structure drawings were obtained by using ORTEPIII [28] and Mercury [29]. CCDC 2390359 and 2435709-2435710 contain the supplementary crystallographic data.

The synthesis and characterization of ligands H_2_L1, H_2_L2, H_2_L3, NaH_2_L4 and NaH_2_L5 and of the copper(II) complexes **1**, **4**, **8** and **9** were previously reported [8,9,10]. Their characterization can be found in the Supporting Information.

Ligands NaH_2_L6 and NaH_2_L7 were obtained by condensation between semicarbazide hydrochloride or octanoic hydrazide and an equimolar amount of 5-sulfonate-2-hydroxy-3-methoxybenzaldehyde sodium salt. The sulfonate aldehyde was synthesized according to procedures in the literature [30,31] as follows. An equimolar amount of aniline in ethanol (5 mL) was added to a solution of 2-hydroxy-3-methoxybenzaldehyde (0.02 mol) in absolute ethanol (20 mL). The mixture was heated at 85 °C for 3 h; the Shiff base intermediate was isolated as an orange powder by evaporating the solvent. It was subsequently reacted with 11 equivalents of concentrated H_2_SO_4_ by heating at 105 °C for 2 h. After cooling in an ice bath, crushed ice was carefully added, obtaining a yellow precipitate, which was filtered off, washed with cold water and recrystallized in the minimum quantity of water. Hydrolysis of the iminic intermediate was performed by suspending the solid (0.01 mol) in 8 mL of water, then a solution of Na_2_CO_3_ (0.014 mol) in water (8 mL) was slowly added. After the complete evolution of CO_2_, the reaction mixture was heated at 105 °C for 2 h, maintaining the round-bottom flask open to facilitate the stripping of the aniline. After cooling to room temperature, the pH was adjusted to 5 by addition of glacial acetic acid. Then, 25 mL of ethanol was added and the solution refrigerated overnight. A precipitate was collected by filtration, which was washed with ethanol and dried in vacuum. Yield: 65%. IR (cm^−1^): ν(C=O) = 1668. ^1^H-NMR (DMSO-*d*_6_, 400 MHz, 25 °C), ppm: 10.23 (s, 1H, CH=O), 7.48 (d, 1H, CH_arom_), 7.31 (d, 1H, CH_arom_); 3.83 (s, 3H, OCH_3_). ^13^C-NMR (DMSO-*d*_6_, 100.5 MHz, 60 °C), ppm: 191.6; 152.9; 148.2; 138.4; 121.1; 117.3; 114.0; 56.0. MS-ESI (negative ions) *m*/*z* (%) = 231.2 ([M − Na]^−^, 100).

**NaH_2_L6∙H_2_O**. 5-Sulfonate-2-hydroxy-3-methoxybenzaldehyde sodium salt (300 mg, 1.1 mmol) was dissolved in 20 mL of methanol and 2 mL of water. An amount of 370 mg (1.1 mmol) of semicarbazide hydrochloride was dissolved in 10 mL of methanol and 1.18 mL of a 1M aqueous solution of NaOH was added; the resulting solution was added to a solution of the aldehyde. The reacting mixture was heated at reflux for 3 h. The solvent was partially evaporated under vacuum and the greenish precipitate was filtered off, washed with cold methanol and dried under vacuum. Yield = 80%.

IR (cm^−1^): ν(C=O) = 1693; ν(C=N) = 1606. ^1^H-NMR (DMSO-*d*_6_, 400 MHz, 25 °C) δ (ppm): 10.24 (s, 1H, OH), 9.61 (s, 1H, NH), 8.17 (s, 1H, HC=N), 7.50 (s, 1H, CH_arom_), 7.14 (s, 1H, CH_arom_); 6.30 (s, 2H, NH_2_); 3.83 (s, 3H, OCH_3_). ^13^C {^1^H} NMR (125 MHz; DMSO-*d*_6_) δ(ppm): 156.78; 147.28; 146.02; 140.04; 138.56; 119.86; 116.09; 110.57; 56.28.

MS-ESI (negative ions) *m*/*z* (%) = 288.2 ([M − Na]^−^, 100). Anal. Calcd. for C_9_H_10_N_3_NaO_6_S∙H_2_O: C 32.83; H 3.67; N 12.76; S 9.90. Found: C 32.81; H 3.72; N 13.07; S 9.90.

**NaH_2_L7∙0.5H_2_O**. The synthetic procedure was as for **NaH_2_L6∙H_2_O** but starting from 218 mg (1.4 mmol) of octanoic hydrazide in 10 mL of methanol and a solution of 5-sulfonate-2-hydroxy-3-methoxybenzaldehyde sodium salt (350 mg, 1.4 mmol) in 20 mL of methanol and 2 mL of water. Yield = 66%. IR (cm^−1^): ν(OH) 3374; ν(N-H) 2926; ν(C=O) = 1677; ν(C=N) = 1606. ^1^H-NMR (DMSO-*d*_6_, 400 MHz, 25 °C) δ (ppm): *E isomer* 11.57 (s, 1H, OH), 11.03 (s, 1H, NH), 8.34 (s, 1H, HC=N), 7.37 (s, 1H, CH_arom_), 7.16 (s, CH_arom_, overlapping isomers); 3.81 (s, OCH_3_, overlapping isomers), 2.21 (t, 2H, α-CH_2_), 1.57 (q, 2H, β-CH_2_, overlapping isomers), 1.27 (m, CH_2_, overlapping isomers), 0.86 (m, CH_3_, overlapping isomers). *Z isomer* 11.18 (s, 1H, OH), 9.62 (s, 1H, NH), 8.26 (s, 1H, HC=N), 7.49 (s, 1H, CH_arom_), 7.15 (s, CH_arom_, overlapping isomers); 3.82 (s, OCH_3_, overlapping isomers), 2.58 (t, 2H, α-CH_2_), 1.57 (q, 2H, β-CH_2_, overlapping isomers), 1.27 (m, CH_2_, overlapping isomers), 0.86 (m, CH_3_, overlapping isomers). ^13^C {^1^H} NMR (125 MHz; DMSO-*d*_6_) δ(ppm): 174.33; 168.91; 147.48; 147.36; 147.34; 147.38; 145.96; 140.27; 140.11; 119.63; 118.41; 118.01; 115.39; 111.26; 110.54; 52.29; 34.40; 32.29; 31.67; 31.61; 29.23; 29.09; 29.08; 28.90; 25.36; 24.54; 22.53; 14.42. MS-ESI (negative ions) *m*/*z* (%) = 371.2 ([M − Na]^−^, 100); 765 ([2M − Na]^−^, 100). Anal. Calcd. for C_16_H_23_N_2_NaO_6_S∙0.5H_2_O: C 47.63; H 6.00; N 6.94; S 7.95. Found: C 47.15; H 5.79; N 7.00; S 7.87.

**(2)** [Ni(HL1)_2_]. H_2_L1 (150 mg, 0.6 mmol) was suspended in 20 mL of ethanol at reflux and Ni(CH_3_COO)_2_ 4H_2_O (80 mg, 0.3 mmol) in 2 mL of the same solvent was added. The brown mixture was heated at reflux for 4 h, then cooled and the solvent partially evaporated by vacuum. The mixture was kept at −20 °C overnight, then the brown-red precipitate was separated by centrifugation, washed with ethanol and dried under vacuum. Yield = 30%. IR(cm^−1^): ν(OH) 3460; ν(NH) 3342; ν(C=N) 1532. ^1^H-NMR (DMSO-*d*_6_, 400 MHz, 25 °C), δ (ppm):12.26 (s, 1H), 9.96 (s, 1H), 9.28 (s, 1H, CH=N), 8.43 (s, 1H, CH=N), 7.91 (s, br, 1H, N_2_); 7.59 (d, br, 1H, CH_arom_); 6.78–7.01 (m, 4H, NH_2_+ CH_arom_); 6.37–6.51 (m, 4H, CH_arom_); 3.81 (s, 3H, OCH_3_), 3.71 (s, 3H, OCH_3_). Anal. Calcd. for C_18_H_20_N_6_O_4_S_2_Ni∙0.5H_2_O: C 41.16, H 4.22, N 16.00, S 12.21. Found: C 41.68, H 3.84, N 15.82, S 12.27.

**(3)** [Zn(HL1)_2_]. H_2_L1 (150 mg, 0.6 mmol) was suspended in 20 mL of methanol at reflux and the pH adjusted to 8–9 with NaOH. Zn(CH_3_COO)_2_ 4H_2_O (73 mg, 0.3 mmol) was dissolved in 2 mL of the same solvent and added to the solution of the ligand. The reacting mixture was refluxed for 6 h, then cooled and the solvent partially evaporated by vacuum. The mixture was kept at −20 °C overnight, then the yellow precipitate was filtered off, washed with cold methanol and dried under vacuum. Yield = 54%. IR(cm^−1^): ν(NH) 3104; ν(C=N) 1598. Anal. Calcd. for C_18_H_20_N_6_O_4_S_2_Zn: C 42.04, H 3.90, N 16.35, S 12.48. Found: C 41.94, H 3.94, N 16.17, S 12.45.

**(5)** [Cu(HL3)Cl]∙0.5H_2_O∙0.5CH_3_OH. H_2_L3 (150 mg, 0.7 mmol) was dissolved in 20 mL of methanol at reflux. CuCl_2_ 2H_2_O (120 mg, 0.7 mmol) was dissolved in 2 mL of the same solvent and added. The dark green solution was heated at reflux for 4 h, then cooled and the solvent partially evaporated by vacuum. A green precipitate formed, which was filtered off, washed with ether and dried under vacuum. Yield = 40%. IR(cm^−1^): ν(NH) 3280–3150; ν(CH) 3025–2966; ν(C=O) 1654; ν(C=N) 1537. MS-ESI (negative ions) *m*/*z* (%) = 305.0 ([CuLCl]^−^, 100). Anal. Calcd. for C_9_H_10_N_3_O_3_CuCl∙0.5H_2_O∙0.5CH_3_OH: C 34.34, H 3.94, N 12.65. Found: C 34.29, H 3.65, N 12.54.

**(6)** [Ni(HL3)_2_]. H_2_L3 (150 mg, 0.70 mmol) was suspended in 20 mL of methanol at reflux and the pH adjusted to 8–9 by adding an aqueous solution of NaOH 1.2M. Ni(CH_3_COO)_2_ 4H_2_O (89 mg, 0.35 mmol) in 2 mL of the same solvent was added. The reacting mixture was heated at reflux for 6 h, then cooled and the solvent partially evaporated by vacuum. The light green precipitate was filtered off, washed with cold methanol and dried under vacuum. Yield = 34%. IR(cm^−1^): ν(NH) 3341; ν(C=O) 1648–1626; ν(C=N) 1597–1560. Anal. Calcd. for C_18_H_20_N_6_O_6_Ni∙2H_2_O: C 42.30, H 4.70, N 16.44. Found: C 42.39, H 4.70, N 16.44.

**(7)** [Zn(HL3)_2_]. The synthetic procedure was as for **6** but starting from H_2_L3 (150 mg, 0.6 mmol) and Zn(CH_3_COO)_2_ 4H_2_O (73 mg, 0.3 mmol). Yield = 64%. IR(cm^−1^): ν(C=O) 1657–1623; ν(C=N) 1556–1596. Anal. Calcd. for C_18_H_20_N_6_O_6_Zn∙1.5H_2_O: C 42.49, H 4.29, N 16.52. Found: C 42.55, H 4.56, N 16.30. Anal. Calcd. for C_18_H_20_N_6_O_4_S_2_Ni∙0.5H_2_O: C 41.16, H 4.22, N 16.00, S 12.21. Found: C 41.68, H 3.84, N 15.82, S 12.27.

**(10)** [NaCu(HL6)Cl]. The synthesis is like that used for **6** but starting from NaH_2_L6 (200 mg, 0.6 mmol) and CuCl_2_ 2H_2_O (110 mg, 0.6 mmol). Yield = 83%. IR(cm^−1^): ν(NH) 3407, 3318; ν(C=O) 1656; ν(C=N) 1543. MS-ESI (negative ions) *m*/*z* (%) = 348.9 ([CuHLCl]ˉ, 90%); 384.9 ([CuClHL]ˉ, 100%); 700.7 ([CuClHL]_2_ˉ 60%). Anal. Calcd. for C_9_H_9_ClCuN_3_NaO_6_S: C 26.41; H 2.22; N 10.27; S 7.84. Found: C 26.20; H 2.45; N 10.05; S 7.77.

**(11)** [Ni(HL6)]_2_∙3.5H_2_O. Ligand NaH_2_L6 (150 mg, 0.5 mmol) was suspended in 15 mL of methanol, then the pH was adjusted to 8–9 by adding an aqueous solution of NaOH 1 M. NiCl_2_ 6H_2_O (117 mg, 0.5 mmol) was dissolved in 3 mL of methanol and 2 mL of water and added to the solution of the ligand. The solution turned green. The reacting mixture was stirred at r.t. for 3 h and the solvent partially evaporated by vacuum. Ethanol was added and a precipitate was obtained, which was isolated by centrifugation, washed with cold ethanol and dried under vacuum. By slow evaporation of the mother liquors of **11**, crystals of [Ni(HL6)(H_2_O)_2_]_2_ [Ni(HL6)(H_2_O)_3_] suitable for X-ray diffraction analysis were obtained. Yield = 57%. IR (cm^−1^): ν(OH+NH) 3346; ν(C=O) 1660; ν(C=N) 1549. Anal. Calcd. for C_9_H_9_N_3_NiO_6_S∙3.5H_2_O: C 26.43; H 3.94; N 10.27; S 7.84. Found: C 26.68; H 3.72; N 9.45; S 7.53.

**(12)** [Zn(HL6)]∙3.5H_2_O. The synthesis is like that used for **6** but starting from NaH_2_L6 (150 mg, 0.5 mmol) and ZnCl_2_ (70 mg, 0.5 mmol). By slow evaporation of the mother liquors of **12**, crystals of [Zn(HL^6^)(H_2_O)_2_] suitable for X-ray diffraction analysis were obtained. Yield = 49%. IR (cm^−1^): ν(C=O) 1691; ν(C=N) 1663. ^1^H-NMR (DMSO-*d*_6_, 400 MHz, 25 °C), δ (ppm):11.19 (s, 1H, NH), 8.13 (s, 1H, CH=N), 6.99–7.07 (m, 4H, NH_2_ + CH_arom_); 3.71 (s, 3H, OCH_3_). Anal. Calcd. for C_9_H_9_N_3_ZnO_6_S∙3.5H_2_O: C 26.00; H 3.88; N 10.11; S 7.71. Found: C 26.33; H 3.62; N 9.82; S 7.65.

**(13)** [NaCu(HL7)Cl]. The synthesis is like that used for **6** but starting from NaH_2_L7 (200 mg, 0.5 mmol) and CuCl_2_ 2H_2_O (85 mg, 0.5 mmol). By slow evaporation of a methanol solution of **13**, crystals of [Cu(HL7)(H_2_O)]_2_ suitable for X-ray diffraction analysis were obtained. Yield = 66%. IR (cm^−1^): ν(OH+NH) 3323 (br); ν(C-H) 2924; ν(C=O) 1625; ν(C=N) 1577. MS-ESI (negative ions) *m*/*z* (%) = 468.0 ([CuHLCl]ˉ, 70%); 866.9 ([CuL]_2_^−^, 100%). Anal. Calcd. for C_16_H_22_ClCuN_2_NaO_6_S∙H_2_O: C 37.65; H 4.74; N 5.49; S 6.28. Found: C 37.79; H 4.77; N 5.44; S 6.33.

**(14)** [NaNi(HL7)Cl]∙2.5H_2_O. NaH_2_L7 (150 mg, 0.4 mmol) was suspended in 15 mL of methanol and 2 mL of water and the pH adjusted to 8–9 by means of NaOH 1 M. When NiCl_2_ 6H_2_O (90 mg, 0.4 mmol) in 5 mL of methanol and 2 mL of water was added, the solution turned yellow-green. The solution was stirred at r.t. for 4 h and then the solvent was partially evaporated by vacuum. Ethanol was added and a precipitate was obtained, which was isolated by centrifugation, washed with cold ethanol and dried under vacuum. Yield = 57%. IR (cm^−1^): ν (OH+NH) 3369 (br); ν (C-H) 2928; ν (C=O) 1603; ν (C=N) 1564. Anal. Calcd. for C_16_H_22_ClNiN_2_NaO_6_S∙2.5H_2_O: C 36.08; H 5.11; N 5.26; S 6.02. Found: C 36.22; H 5.08; N 5.06; S 6.22.

### 3.2. Metal Complexes and Reference Drugs

The dry powder of the metal complexes was dissolved in DMSO at 20 mM and then diluted with the appropriate cell culture medium to achieve the required concentrations. Ciprofloxacin, fluconazole and cisplatin, purchased from Sigma-Aldrich (St. Louis, MO, USA), were used as reference drugs in the biological assays.

### 3.3. Biological Evaluation

#### 3.3.1. Microbial Strains and Growth Conditions

Reference strains of *Staphylococcus aureus* (ATCC 25923), *Escherichia coli* (ATCC 25922) and *Candida albicans* (ATCC 10231) were used in this study as model systems. These laboratory strains were purchased from the American Type Culture Collection (ATCC) (Manassas, VA, USA). The cultures were routinely grown on 5% blood agar plates or Sabouraud Dextrose agar plates (Biolife Italiana S.r.l., Milan, Italy) and used freshly in the assays.

#### 3.3.2. MIC and IC_50_ Determination

The antimicrobial properties of the metal complexes were determined by a previously established microdilution method [20,22] in agreement with the Clinical and Laboratory Standard Institute (CLSI) guidelines. In short, colonies obtained on 5% blood agar plates or Sabouraud Dextrose agar plates were used to prepare microbial suspensions (at 0.5 McFarland) and diluted 1:200 in Mueller–Hinton broth (Sigma-Aldrich, St. Louis, MO, USA) for the antibacterial assays and 1:20 in RPMI-1640 medium (Gibco^®^, ThermoFisher Scientific Inc., Waltham, MA, USA) containing glucose 2% and 0.3% levo-glutamine buffered to pH 7.0 with 0.165 M 3-(N-morpholino)propanesulfonic acid (MOPS) for the antifungal assays. The metal complexes were two-fold serially diluted and tested in the range of 100–0.19 µM. Positive controls (in regular media), negative controls (only compounds), solvent controls (DMSO dilutions) and the reference drugs were included in the tests. The plate was incubated at 37 °C for 24 h, and the OD_630nm_ was measured. MIC values were defined as the concentration of the compounds inhibiting the 90% of microbial growth relative to the positive controls and IC_50_ values as the concentration giving rise to an inhibition of growth of 50%, obtained from nonlinear regression analysis (GraphPad Prism version 9.4.1, San Diego, CA, USA).

#### 3.3.3. Cell Viability and Proliferation Assay

HEL 299 cells, obtained from the American Type Culture Collection (ATCC-CCL-137), were selected to investigate the effect of the metal complexes on non-malignant human fibroblasts. Briefly, cells were cultured in Eagle’s Minimum Essential Medium (EMEM) supplemented with 10% fetal bovine serum (FBS), 100 U/mL penicillin and 100 µg/mL streptomycin at 37 °C with 5% CO_2_. For the biological assays, cells were grown in 96-well plates at 10^4^ cells/well. After 24 h of incubation at 37 °C, the monolayer was treated with 100 µL of medium containing the 2-fold serial dilutions of the compounds in the range 100–0.19 µM. Cell viability was assessed by a colorimetric WST8-based assay according to the manufacturer’s instructions (CCK-8, Cell Counting Kit-8, Dojindo Molecular Technologies, Rockville, MD, USA) by measuring the OD at 450 nm. Data are expressed as the percentage of cell viability relative to the untreated controls. The CC_50_ was obtained on the corresponding dose–response curves generated as previously reported for IC_50_ values.

#### 3.3.4. Hemolytic Activity Assay

The hemolytic activity of the metal complexes was evaluated as the amount of hemoglobin released by the disruption of human red blood cells (hRBCs). Briefly, a suspension of hRBCs (4% *w*/*v* in PBS) was prepared from the peripheral blood of anonymous blood donors available for research purposes. Aliquots of 100 µL of the obtained suspension were incubated with an equal volume of the 2-fold dilutions (range 100–0.19 µM) of the compounds for 1 h at 37 °C. Then, the supernatants were spectrophotometrically evaluated at OD_405nm_. Untreated hRBCs (in PBS) and hRBCs incubated with 1% Triton X-100 were employed as negative and positive controls, respectively. The hemolysis percentage was calculated as [OD_405nm_ (sample) − OD_405nm_ (negative control)]/[OD_405nm_ (positive control) − OD_405nm_ (negative control)] × 100. Minimal hemolytic concentrations (MHCs) were defined as the compound concentration causing 10% hemolysis.

### 3.4. In Vitro DNA Cleavage/Mobility Shift Assays

Studies of the interactions between the metal compounds and plasmid DNA were carried out by agarose gel electrophoresis. Thus, 200 ng of pmaxGFP plasmid, 3527 bps, (Lonza, Basel, Switzerland) were incubated with the metal compounds at 100 μM in the absence or presence of 10 mM of H_2_O_2_ in Tris-HCl buffer (NaCl 50 mM, Tris-HCl 5 mM, pH 7.20). The mixture was incubated at 37 °C for 2 h, then the reactions were quenched by adding 5 μL of the loading buffer solution and analyzed by 1% agarose gel electrophoresis.

## 4. Conclusions

In this work, ligands containing a common 2-methoxysalicyl ring were used, differing them by the presence or absence of a sulfonic acid group (SO_3_^−^) and featuring coordinating atom sets of either ONO (semicarbazone/hydrazone) or ONS (thiosemicarbazone). We undertook a screening of the antibacterial (*S. aureus* and *E. coli*) and antifungal (*C. albicans*) properties of some copper(II) (**1**, **4**, **5**, **8**–**10**, **13**), nickel(II) (**2**, **6**, **11**, **14**) and zinc(II) (**3**, **7**, **12**) complexes with these ligands. We selected these microorganisms as representative strains for bacteria and fungi; they are all pathogens responsible for a variety of community- and hospital-acquired infections and characterized by increasing antimicrobial resistance to many classes of antimicrobial agents. These microorganisms have become a major concern in global public health, invigorating the need for new antimicrobial compounds.

Unfortunately, none of the studied complexes showed appreciable activity against *C. albicans* (only 4 had a modest IC_50_ = 54.8 μM) or the Gram-negative bacterium *E. coli*, while the copper complexes 1 and 4 and the nickel complex **2** showed micromolar activity against the Gram-positive bacterium *S. aureus*. It should be noted that **1**, **2** and **4** are all with *ONS* thiosemicarbazone ligands without the sulfonic group: no complex containing an *ONO* ligand is significantly active, just as no complex with a sulfonate ligand is active, regardless of whether it is *ONO* or *ONS*. If we analyze the role of the ligand, evidently, the C=S group is fundamental for the activity. The sulfonic group SO_3_^−^ makes the complexes soluble in an aqueous environment, but its negative charge probably affects interaction with the bacterial membrane, preventing the transport of the metal ion inside the cell. If we analyze the data from the point of view of the metal ion used, it can be observed that the zinc(II) complexes are not active in any case, while the most active against *S. aureus* are the two copper(II) complexes **1** and **4** (IC_50_ = 4.2 μM and 3.5 μM, respectively) and, to a lesser extent, the nickel(II) complex 2 (61.8 μM). However, it is important to note that **2** has an SI of 1.2, calculated as the ratio between CC_50_ in HEL 299 and IC_50_. This finding is of biological relevance because it indicates the preferential inhibitory effect of the complex on the bacterial target rather than on human fibroblasts. In addition, the nickel(II) complex **2** did not exert a hemolytic effect on hRBCs, thus confirming its safety in mammalian cells.

The complexes were used in an in vitro DNA cleavage assay in the absence and presence of H_2_O_2_, attempting to clarify their mechanisms of action. In absence of H_2_O_2_, no complex was active, while in the presence of H_2_O_2_, all the copper(II) complexes, except 13, degraded plasmid DNA. It is therefore possible to conclude that the antibacterial activity of 1 and 4 may be, at least partially, linked to the production of free radicals. Zinc(II) complexes do not degrade DNA, and in fact, the zinc(II) ion is not, unlike the copper(II) ion, a good catalyst for ROS production. We also believe that the lack of antibacterial activity of copper complexes **5** and **8–10**, which also degrade plasmid DNA, may be linked to their inability to cross the cell barrier. The challenge of crossing the cell membrane is even greater in the case of Gram-negative bacteria: in this case, none of the complexes are active. Supporting this observation, only nickel(II) complex **2**—the sole complex with antibacterial activity— was able to promote plasmid DNA cleavage and, even then, only in the presence of H_2_O_2_. However, unlike the copper(II) complexes, only one strand of the plasmid DNA is cleaved, leading to the formation of the open circular form: this suggests that also complex **2** leads to the production of ROS but the catalytic activity of the nickel(II) complex is less effective [32].

## Figures and Tables

**Table 1:** Percentage values of microbial growth at 100 μM (mean and standard deviation).

Compound	*S. aureus*	*E. coli*	*C. albicans*
**1**	0.3 ± 0.5	88.9 ± 8.3	43.6 ± 6.1
**2**	3.1 ± 4.3	86.1 ± 24.4	77.1 ± 6.0
**3**	102.3 ± 6.7	106.1 ± 4.3	124.3 ± 3.6
**4**	0.3 ± 0 5	110.5 ± 4.8	15.6 ± 5.5
**5**	83.6 ± 1.4	55.8 ± 0.5	77.8 ± 2.0
**6**	97.7 ± 3.0	101.9 ± 1.8	130.8 ± 3.6
**7**	107.0 ± 2.7	82.0 ± 3.2	121.4 ± 4.4
**8**	106.3 ± 1.6	104.1 ± 9.3	89.9 ± 5.3
**9**	104.8 ± 0.7	96.6 ± 1.1	91.4 ± 2.5
**10**	97.8 ± 1.4	99.8 ± 1.8	71.6 ± 1.3
**11**	90.0 ± 1.1	99.4 ± 0.8	82.7 ± 2.7
**12**	94.2 ± 0.9	108.0 ± 3.9	89.1 ± 3.8
**13**	99.8 ± 3.6	105.2 ± 2.0	81.7 ± 5.4
**14**	97.7 ± 5.3	113.6 ± 10.0	85.8 ± 3.4
CIP ^1^	0.7 ± 0.6	0.2 ± 0.3	n.d. ^2^
FCZ ^3^	n.d.	n.d.	10.0 ± 0.6
DMSO ^4^	100 ± 2.5	100 ± 4.4	100 ± 1.5

^1^ CIP, ciprofloxacin at 0.6 μM; ^2^ n.d., not determined; ^3^ FCZ, fluconazole at 1.6 μM; ^4^ DMSO, dimethyl sulfoxide at 0.5%.

**Table 2:** Percentage values of cell viability and hemolytic activity at 100 μM (mean and standard deviation).

Compound	HEL 299	hRBCs
**1**	3.0 ± 3.3	<5
**2**	26.5 ± 5.8	<5
**3**	68.5 ± 7.1	<5
**4**	9.1 ± 1.1	<5
**5**	20.3 ± 0.6	<5
**6**	54.1 ± 8.0	<5
**7**	55.3 ± 6.2	<5
**8**	90.4 ± 0.6	<5
**9**	85.8 ± 1.7	<5
**10**	63.5 ± 3.8	<5
**11**	87.3 ± 2.3	<5
**12**	88.2 ± 3.5	<5
**13**	88.7 ± 0.9	<5
**14**	83.9 ± 0.8	<5
CP ^1^	9.2 ± 3.8	n.d. ^2^
Triton X-100 ^3^	n.d.	100 ± 2.6

^1^ CP, cisplatin at 166.6 μM; ^2^ n.d., not determined; ^3^ Triton X-100 at 1%.

**Table 3:** IC_50_ and CC_50_ values (mean values and 95% Confidence Interval) expressed in μM.

Compound	*S. aureus*, IC_50_	*C. albicans*, IC_50_	HEL 299, CC_50_	SI ^1^
**1**	4.2 [3.7–4.7]	n.d. ^2^	2.0 [1.7–2.3]	0.5
**2**	61.8 [50.9–75.1]	n.d.	73.1 [58.1–92.0]	1.2
**4**	3.5 [2.4–5.1]	54.8 [31.8–74.3]	1.4 [1.3–1.6]	0.4 ^3^/0.02 ^4^

^1^ SI, Selectivity Index (CC_50_/IC_50_); ^2^ n.d., not determined; ^3^ S.I. for *S. aureus;* ^4^ SI for *C. albicans.*

## Data Availability

Full crystallographic data has been deposited with the CCDC (2390359, 2435709, and 2435710) and can be obtained free of charge via https://www.ccdc.cam.ac.uk/conts/retrieving.html (accessed on 9 April 2025 or from the Cambridge Crystallographic Data Centre, 12 Union Road, Cambridge CB2 1EZ, UK; fax: (+44) 1223-336-033 or e-mail: deposit@ccdc.cam.ac.uk.

## Supplementary Materials

The following supporting information can be downloaded at: https://www.mdpi.com/article/10.3390/molecules30112329/s1. Scheme S1. Synthesis of the ligands. Semicarbazide is used as chlorohydrated salt and aqueous NaOH is added in situ to obtain the neutral semicarbazide. Figure S1. (A) 1H NMR (top) and 13C NMR (DEPT, bottom) spectrum of NaH2L6 registered in DMSO-d6. (B) ATR-IR spectrum and (C) mass spectrum (ESI-MS, negative ions) of ligand NaH2L6. Figure S2. (A)1H NMR (top) and 13C NMR (DEPT, bottom) spectrum of NaH2L7 registered in DMSO-d6. (B) ATR-IR spectrum and (C) mass spectrum (ESI-MS, negative ions) of ligand NaH2L6. Figure S3. IR spectrum of complex 5. Figure S4. ESI-MS spectrum for complex 5 (negative ions, solvent: methanol). Figure S5. IR spectrum of complex 2. Figure S6. IR spectrum of complex 3. Figure S7. IR spectrum of complex 6. Figure S8. IR spectrum of complex 7. Figure S9. 1H NMR (top) and 13C NMR (bottom) spectrum of complex 2 registered in DMSO-d6. Figure S10. 1H NMR spectrum of complex 7 registered in DMSO-d6. Figure S11. 1H NMR spectrum of complex 12 registered in DMSO-d6. Figure S12. 1H NMR spectrum of complex 14 registered in DMSO-d6. Figure S13. IR spectrum of complex 10. Figure S14. IR spectrum of complex 11. Figure S15. IR spectrum of complex 12. Figure S16. IR spectrum of complex 13. Figure S17. IR spectrum of complex 14. Figure S18. ESI-MS spectrum for complex 10 (negative ions; solvent: methanol). Figure S19. ESI-MS spectrum for complex 13 (negative ions; solvent: methanol). Figure S20. Cisplatin binding interaction with plasmid DNA. Figure S21. ORTEP representation of [Ni(HL6)(H2O)2]2[Ni(HL6)(H2O)3]. Figure S22. ORTEP representation of [Zn(HL6)(H2O)2]. Figure S23. ORTEP representation of the head-to-tail dimers [Cu(HL7)(H_2_O)]2 (left) and of the molecular unit [Cu(HL7)(H2O)]. Figure S24. UV-visible spectra for compound 13 (0.25 μM) dissolved in a 25 mM HEPES/NaCl (0.9%) solution at pH 7.4 over 72 h. Table S1. Microbial growth and cell proliferation at 100 μM (mean values and standard deviations). Table S2. X-ray crystallographic data for compound [Ni(HL6)(H2O)_2_]_2_[Ni(HL6)(H_2_O)_3_]. Table S3. X-ray crystallographic data for compound 12. Table S4. X-ray crystallographic data for compound [Cu(HL7)(H2O)].
