# Supplementary material for: Antimicrobial Activity of Copper(II), Nickel(II) and Zinc(II) Complexes with Semicarbazone and Thiosemicarbazone Ligands Derived from Substituted Salicylaldehydes
Source: Molecules. 2025 May 26;30(11):2329. doi: 10.3390/molecules30112329 (PMC12155845; doi:10.3390/molecules30112329)

Supporting Information

2-Hydroxy-3-methoxybenzylidene thiosemicarbazide (H_2L^1). White powder. Yield = 71 %. ^{1}H-NMR (DMSO-d6, 25 °C), *δ*: 3.80 (*s*, 3H, OCH_3), 6.77 (t, 1H; *J* = 7.9 Hz, ArH), 6.96 (d, 1H, *J* = 7.8 Hz, ArH), 7.52 (d, 1H, *J* = 7.8 Hz, ArH), 7.87 (s, br, 1H, NH), 8.09 (s, br, 1H, NH), 8.40 (s, 1H; HC=N), 9.20 (s, br, 1H, OH), 11.39 (s, br, 1H; NH). ESI-MS (positive ions, m/z) = 225.0 ([M]+, 100); IR (ATR, cm^{-1}): *ν*NH = 3459, 3339; *ν*OH = 3158 (br); *ν*C=N = 1596; *ν*C=S = 1532, 821; *ν*OCH_3 = 1258, 1056. Anal. Calcd. for $C_9H_{11}N_3O_2S{\cdot}0.5H_2O$: C 46.14; H 5.16; N 17.94. Found: C 45.92, H 4.82, N 18.24.

2-hydroxy-3-methoxybenzaldehyde-4-ethyl-3-thiosemicarbazone (H_2L^2). White powder. Yield = 85%. ^{1}H-NMR (DMSO-d_6, 25°C), δ: 1.13 (t, 3H, $CH_2\underline{CH_3}$); 3.58 (q, 2H, $\underline{CH_2}CH_3$); 3.81 (s, 3H, OCH_3); 6.78 (t, 1H, ArH, J = 7.9); 6.96 (d, 1H, ArH, J = 6.6); 7.54 (d, 1H, ArH, J = 7.3); 8.39 (s, 1H, CH=N); 8.44 (s, br, 1H, NH); 9.20 (s, br, 1H, OH); 11.38 (s, 1H, NH). ESI-MS (positive ions, m/z) = 253.1 ($[M]^+$, 100). IR (ATR, cm^{-1}): νNH = 3305; νC=N = 1522; νC=S= 1063, 784. Anal. Calcd. for $C_{11}H_{15}N_3O_2S{\cdot}H_2O$: C 48.69, H 6.13, N 15.49, S 11.82. Found: 48.66, H 6.25, N 15.54, S 12.01.

2-hydroxy-3-methoxybenzylidene semicarbazone (H_2L^3). Yield = 81%. ^{1}H-NMR (DMSO-d_6, 25°C), δ: 3.84 (s, 3H, OCH_3), 6.39 (s, br, 2H, NH_2), 6.76 (t, J = 7.8 Hz, 1H, ArH), 6.92 (d, J = 7.6 Hz, 1H, ArH), 7.37 (d, 1H; J = 7.8, ArH), 8.17 (s, 1H; HC=N), 9.20 (s, br, 1H; OH), 10.20 (s, br, 1H; NH). ESI-MS (positive ions, m/z) = 209.2 ($[M]^+$, 100); IR (ATR; cm^{-1}): νNH = 3466; νOH = 3170-3280 (br); νC=O = 1672; νC=N = 1586. Anal. Calcd for $C_9H_{11}N_3O_3{\cdot}1/4H_2O$: C 50.58; H 5.42; N 19.66. Found: C 50.67; H 5.37; N 19.44.

Sodium 2-hydroxy-3-methoxy-5-sulfonate-benzaldehyde-3-thiosemicarbazone (NaH_2L^4). Beige powder. Yield = 53%. ^{1}H NMR (DMSO-d_6, 25°C), δ: 11.41 (b, 1H, NH), 9.38 (b, 1H, OH), 8.40 (s, 1H, CH=N), 8.11 (b, 1H, NH), 7.71 (b, 1H, NH), 7.60 (d, *J* = 1.9 Hz, 1H, ArH), 7.16 (d, *J* = 1.9 Hz, 1H, ArH), 3.81 (s, 3H, OCH_3). ^{13}C NMR (101 MHz, DMSO-d_6, 25°C), δ: 177.7, 146.9, 146.2, 140.5, 139.7, 119.1, 115.6, 110.4, 55.9. ESI-MS (negative ions, m/z): 304.3 $(M\text{-}Na)^-$. IR (ATR, cm^{-1}): νNH_2+νOH = 3384; νNH =

3173; νC=N = 1609; νC=S = 1257; $νSO_3$ = 1104, 1038. Anal. Calcd for $C_9H_{10}N_3NaO_5S_2·H_2O$: C 31.30, H 3.50, N 12.17, S 18.57. Found: C 31.56, H 3.17, N 12.17, S 18.98.

Sodium 2-hydroxy-3-methoxy-5-sulfonate-benzaldehyde-4-ethyl-3-thiosemicarbazone (NaH_2L^5). White powder. Yield = 57%. ^{1}H NMR (DMSO-d$_6$, 25°C), δ: 11.35 (s, 1H, NH), 9.37 (b, 1H, OH), 8.41 (s, 1H, CH=N), 8.33 (t, *J* = 6.0 Hz, 1H, NH), 7.63 (d, *J* = 1.9 Hz, 1H, ArH), 7.17 (d, *J* = 1.9 Hz , 1H, ArH), 3.81 (s, 3H, OCH_3), 3.58 (p, *J* = 6.9 Hz, 2H, CH_2), 1.14 (t, *J* = 7.1 Hz, 3H, CH_3). ^{13}C NMR (101 MHz, DMSO-d$_6$, 25°C), δ: 176.5, 146.8, 146.1, 140.0, 139.6, 119.4, 115.6, 110.4, 55.9, 18.6, 14.7. ESI-MS (negative ions, m/z): 332.2 $(M-Na)^-$. IR (ATR, cm^{-1}): νOH = 3384; νNH = 3173; νC=N = 1609; νC=S = 1257; $νSO_3$ = 1104, 1038. Anal. Calcd for $C_{11}H_{14}N_3S_2O_5Na$: C 37.17, H 3.97, N 11.82, S 18.05. Found: C 37.08, H 4.30, N 11.19, S 18.11.

General synthesis for complexes 1, 4, 5, 8, 9. 0.15 g of the ligand were dissolved in methanol and the pH was adjusted to 8-9 by adding NaOH 1M, resulting in a yellow solution. An equimolar amount of $CuCl_2·2H_2O$ in methanol was added and a dark green precipitate appeared. The suspension was stirred for 4 hours at r.t. The solvent was partially evaporated under vacuum and the dark green powder was filtered off and washed with cold methanol.

(1), $[CuL^1Cl]·3H_2O$. Dark green powder. Yield: 52%. MS-ESI (positive ions) m/z (%) = 287 ($[CuL]^+$, 100); 345 ($[CuLCl+Na]^+$, 70). IR (ATR, cm^{-1}): νNH= 3280, 3168; νC=N+δN-H = 1634, 1604; νCS= 1217. Anal. Calcd. for $C_9H_{10}CuClN_3O_2S·3H_2O$: C 28.65; H 4.27; N 11.14. Found: C 28.82; H 4.09; N 10.96.

(4), $[CuL^2Cl]·1.5\ H_2O$. Dark green powder. Yield: 71%. MS-ESI (positive ions) m/z (%) = 315 ($[CuL]^+$, 100); 373 ($[CuLCl+Na]^+$, 10). IR (ATR, cm^{-1}): νNH = 3347, 3299; νC=N+δN-H= 1605, 1586; νCS= 1219. Anal. Calcd. for $C_{11}H_{14}N_3O_2SCuCl·1.5\ H_2O$: C 34.92, H 4.53, N 11.11. Found: C 34.84, H 3.98, N 10.87.

(8), $[Cu(NaHL^4)Cl]$. Dark green powder. Yield: 77%. MS-ESI (negative ions) m/z (%) = 365.2 ($[CuL]^-$, 100). IR (ATR, cm^{-1}): νOH+$νNH_2$ = 3433, 3317; νC=N = 1650; νC=S = 1248; $νSO_3$ = 1170,

1040. Anal. Calcd. for $C_9H_9N_3NaO_5S_2CuCl$: C 25.42, H 2.13, N 9.88, S 15.08. Found: C 25.23, H 2.26, N 9.49, S 15.47.

(9), $[Cu(NaHL^5)Cl]\cdot H_2O$. Dark green powder. Yield: 90%. MS-ESI (negative ions) m/z (%) = 393.3 ($[CuL]^-$, 100); 789.0 ($([Cu(HL)]_2\text{-}H^+)^-$, 10). IR (ATR, cm^{-1}): νNH = 3154, 3053; νC=N= 1606; νC=S = 1245; νSO_3 = 1176, 1036. Anal. Calcd. for $C_{11}H_{13}N_3NaO_5S_2CuCl\cdot H_2O$: C 28.03, H 3.21, N 8.91, S 13.61. Found: C 27.89, H 3.20, N 8.65, S 14.01.

Scheme S1. Synthesis of the ligands. Semicarbazide is used as chlorohydrated salt and aqueous NaOH is added in situ to obtain the neutral semicarbazide.

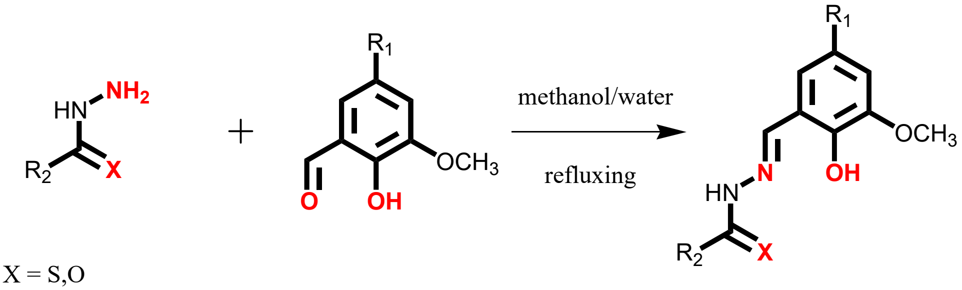

X = S,O

R2 = NH_2, NHEt, NHC_7H_{15}

R_1 = H, HSO_3^-

Figure S1. A)^{1}H NMR (top) and ^{13}C NMR (DEPT, bottom) spectrum of NaH_2L6 registered in DMSO-d_6. **B)** ATR-IR spectrum and **C)** mass spectrum (ESI-MS, negative ions) of ligand NaH_2L6.

A)

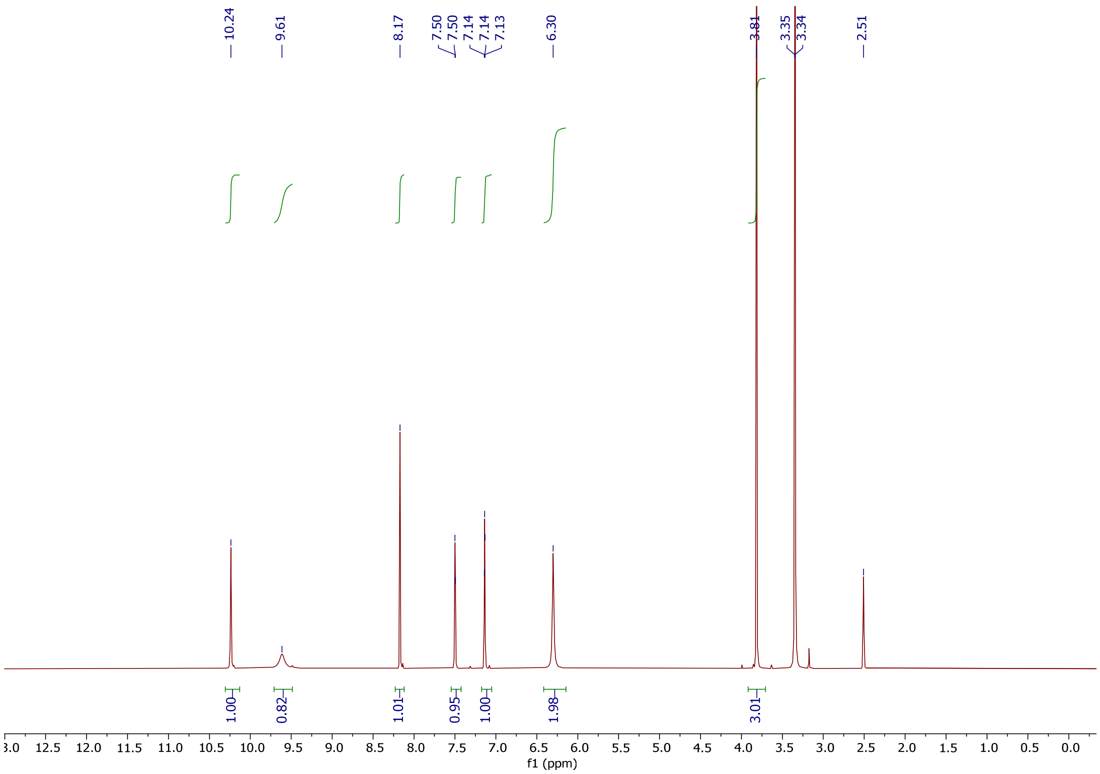

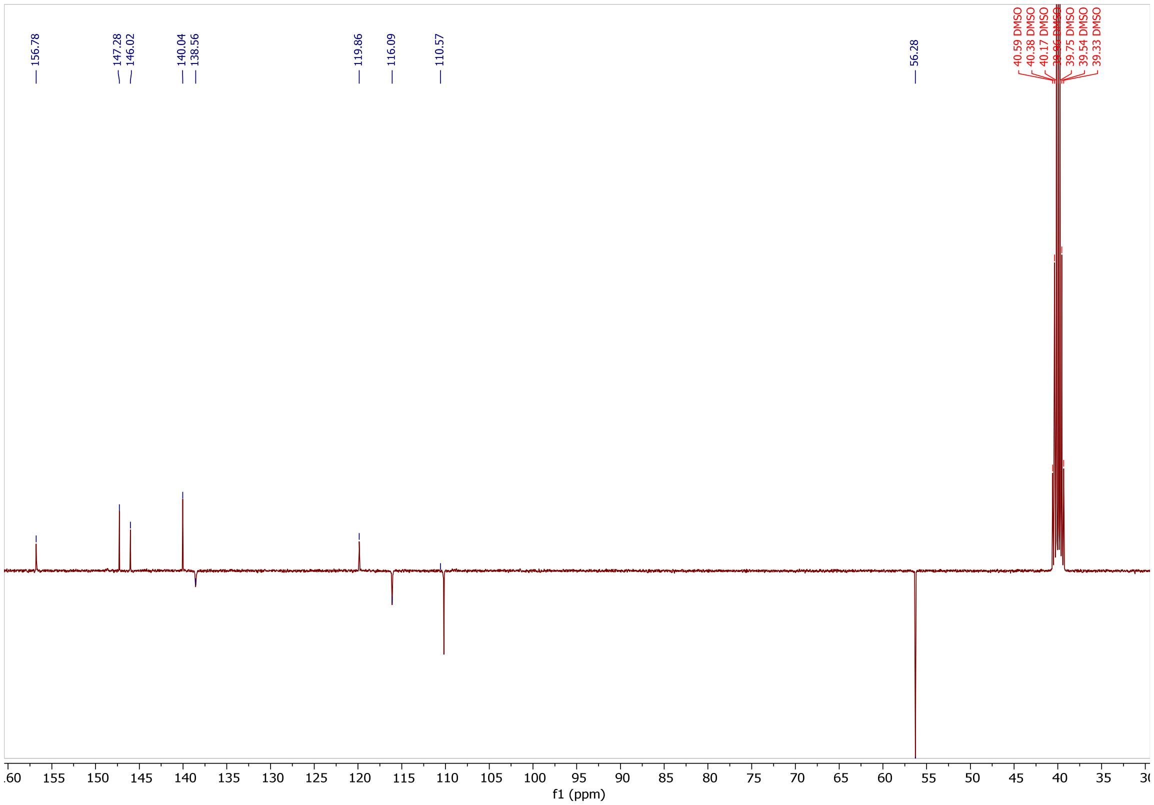

B)

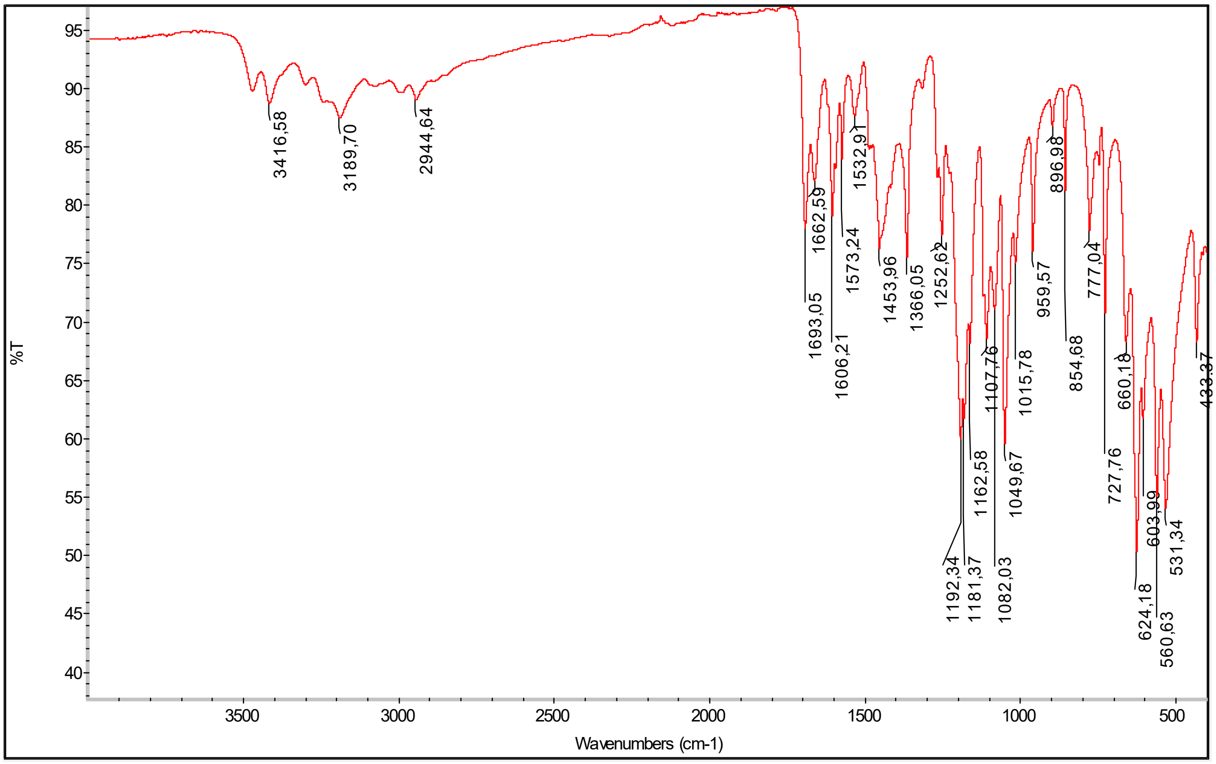
%T
Wavenumbers (cm-1)
3416,58
3189,70
2944,64
1693,05
1662,59
1606,21
1573,24
1532,91
1453,96
1366,05
1252,62
1192,34
1181,37
1162,58
1107,76
1082,03
1049,67
1015,78
959,57
896,98
854,68
777,04
727,76
660,18
624,18
603,99
560,63
531,34
433,37

C)

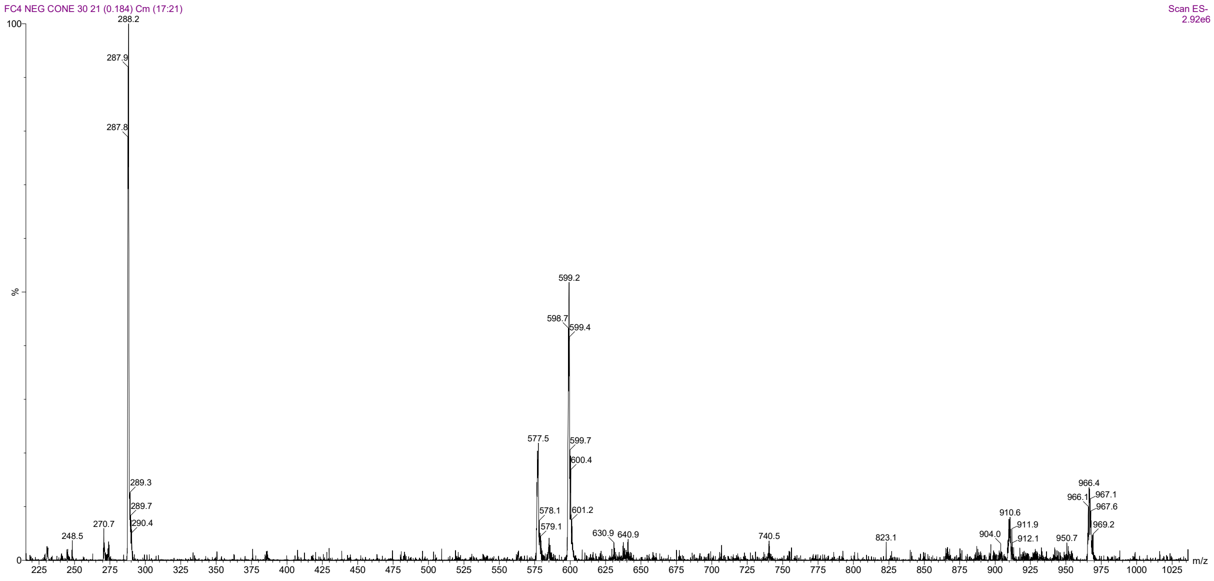
FC4 NEG CONE 30 21 (0.184) Cm (17:21)
Scan ES-
2.92e6
%
m/z
288.2
287.9
287.8
289.3
289.7
290.4
270.7
248.5
599.2
598.7
599.4
577.5
599.7
600.4
578.1
579.1
601.2
630.9
640.9
740.5
823.1
904.0
910.6
911.9
912.1
950.7
966.4
966.1
967.1
967.6
969.2

Figure S2. A)¹H NMR (top) and ¹³C NMR (DEPT, bottom) spectrum of NaH_2L7 registered in DMSO-d_6. **B)** ATR-IR spectrum and **C)** mass spectrum (ESI-MS, negative ions) of ligand NaH_2L6.

A)

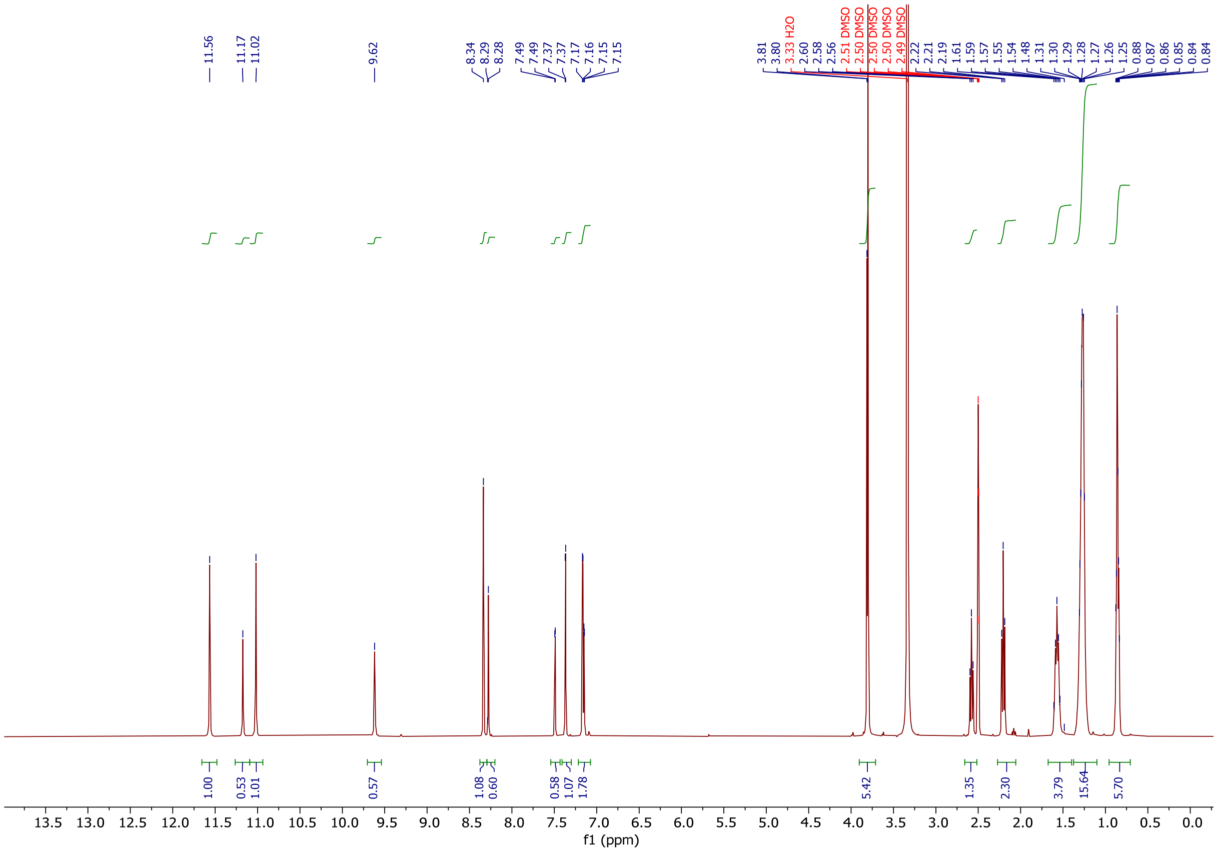

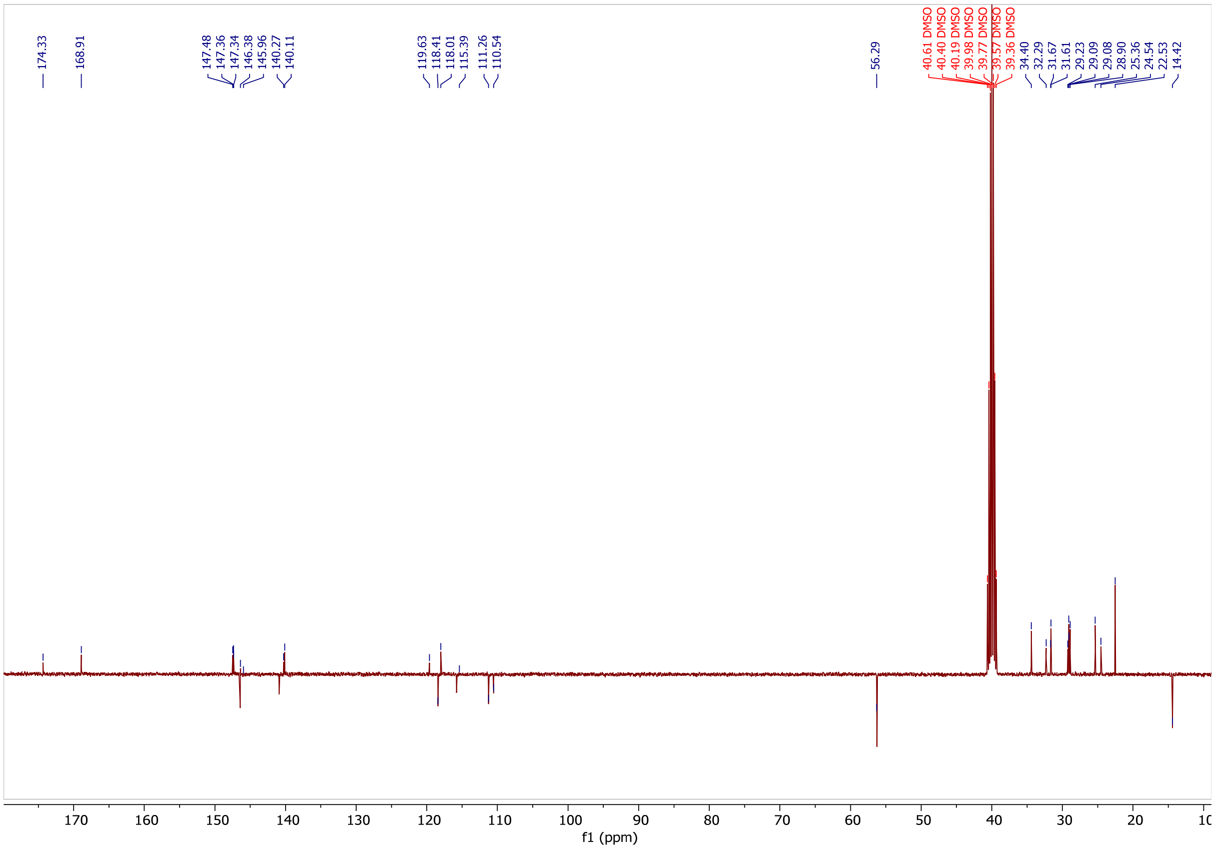

174.33
168.91
147.48
147.36
147.34
146.38
145.96
140.27
140.11
119.63
118.41
118.01
115.39
111.26
110.54
56.29
40.61 DMSO
40.40 DMSO
40.19 DMSO
39.98 DMSO
39.77 DMSO
39.57 DMSO
39.36 DMSO
34.40
32.29
31.67
31.61
29.23
29.09
29.08
28.90
25.36
24.54
22.53
14.42
f1 (ppm)

B)

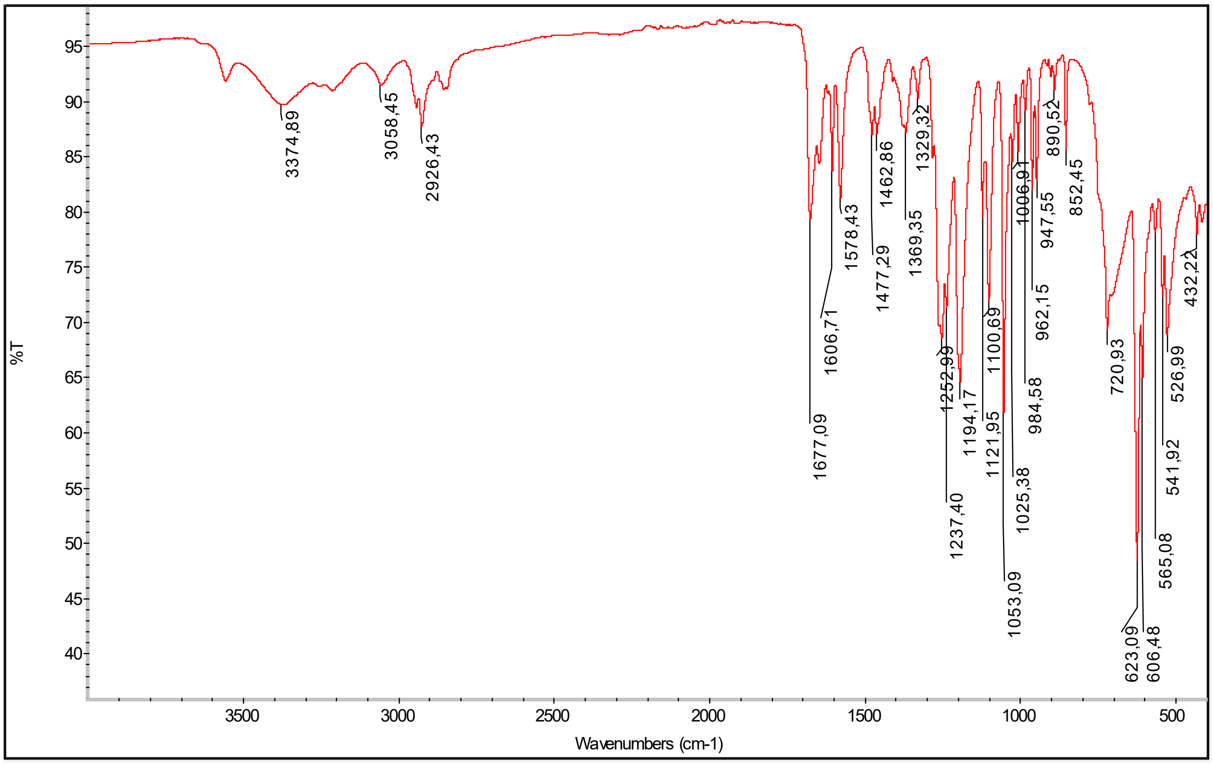

3374,89
3058,45
2926,43
1677,09
1606,71
1578,43
1477,29
1462,86
1369,35
1329,32
1252,99
1237,40
1194,17
1121,95
1100,69
1053,09
1025,38
1006,91
984,58
962,15
947,55
890,52
852,45
720,93
623,09
606,48
565,08
541,92
526,99
432,22
%T
Wavenumbers (cm-1)

C)

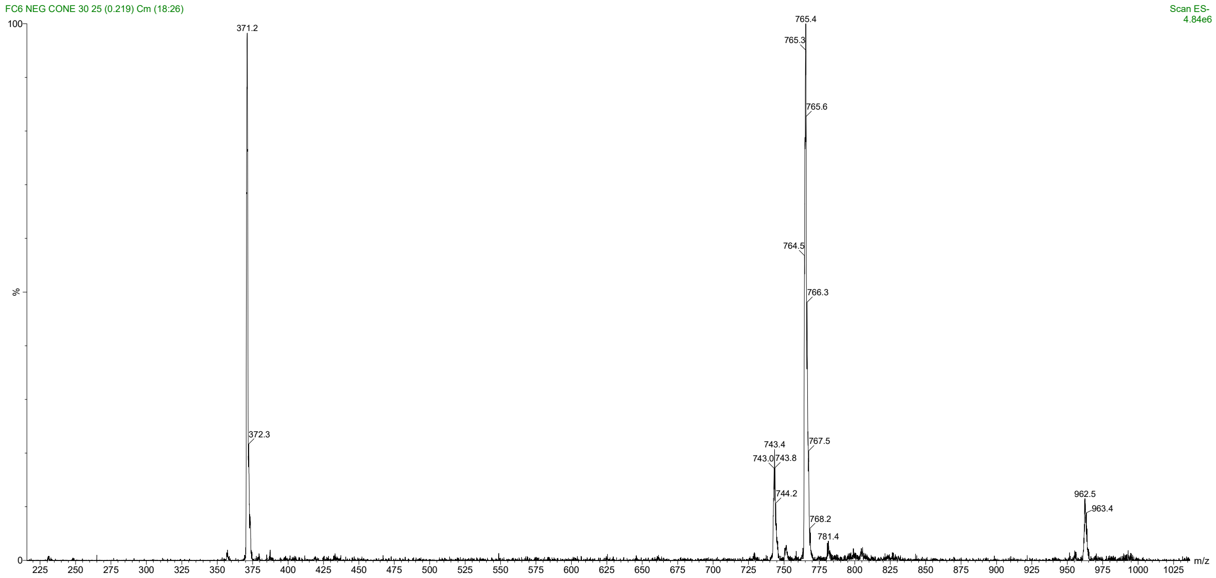
FC6 NEG CONE 30 25 (0.219) Cm (18:26)
Scan ES-
4.84e6
100
%
0
371.2
372.3
765.4
765.3
765.6
764.5
766.3
767.5
743.4
743.0
743.8
744.2
768.2
781.4
962.5
963.4
225
250
275
300
325
350
375
400
425
450
475
500
525
550
575
600
625
650
675
700
725
750
775
800
825
850
875
900
925
950
975
1000
1025
m/z

Figure S3. IR spectrum of complex **5**.

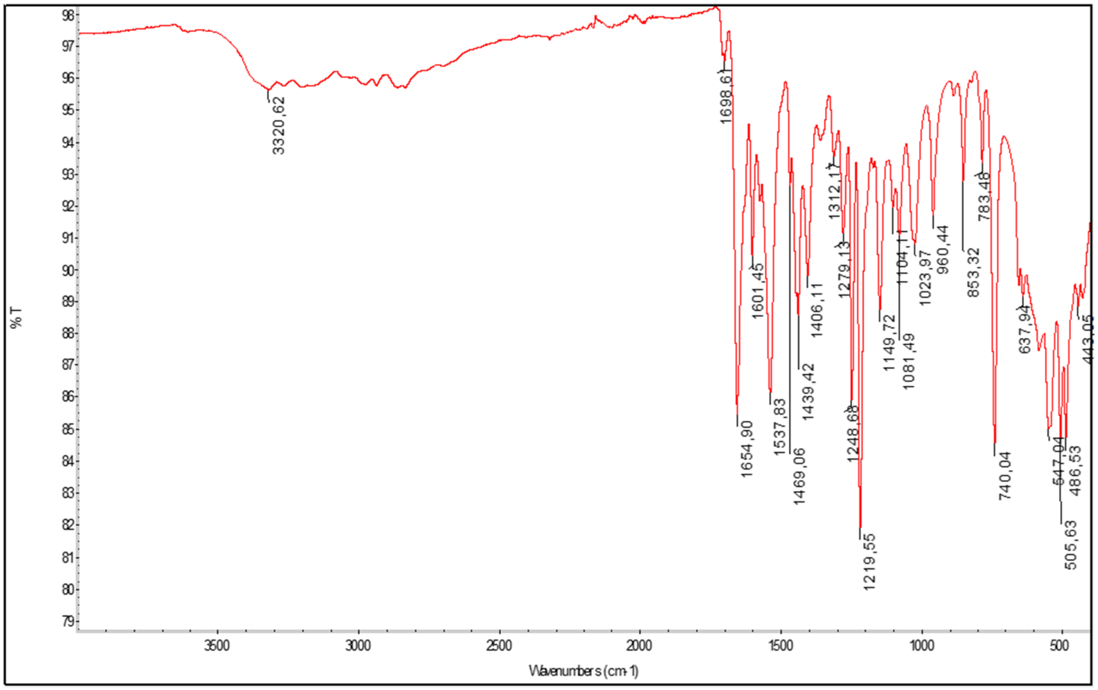

Figure S4. ESI-MS spectrum for complex **5** (negative ions, solvent: methanol).

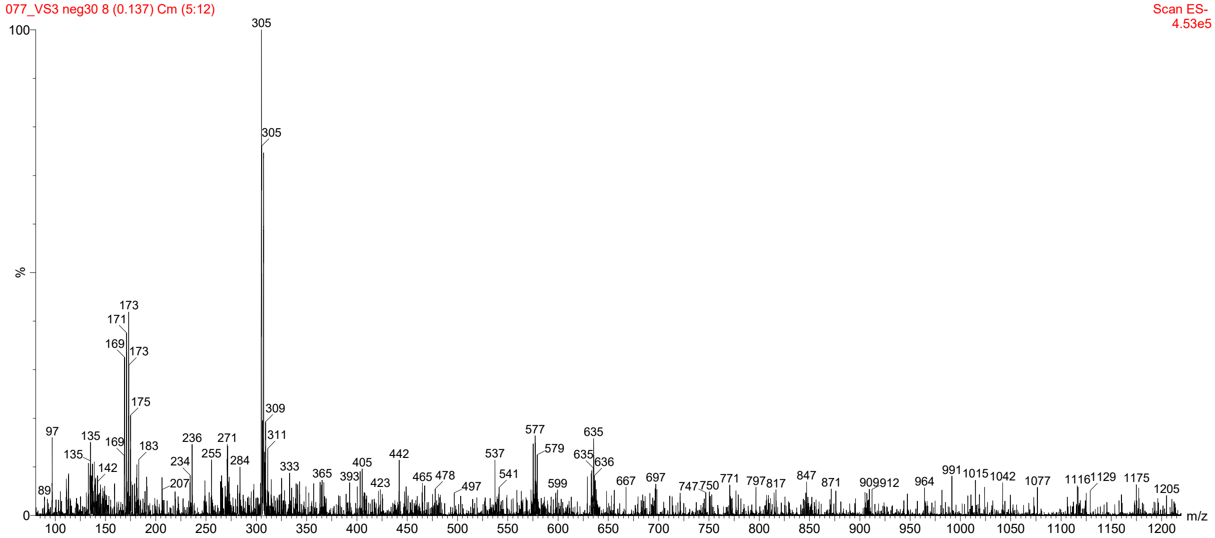

Figure S5. IR spectrum of complex **2**.

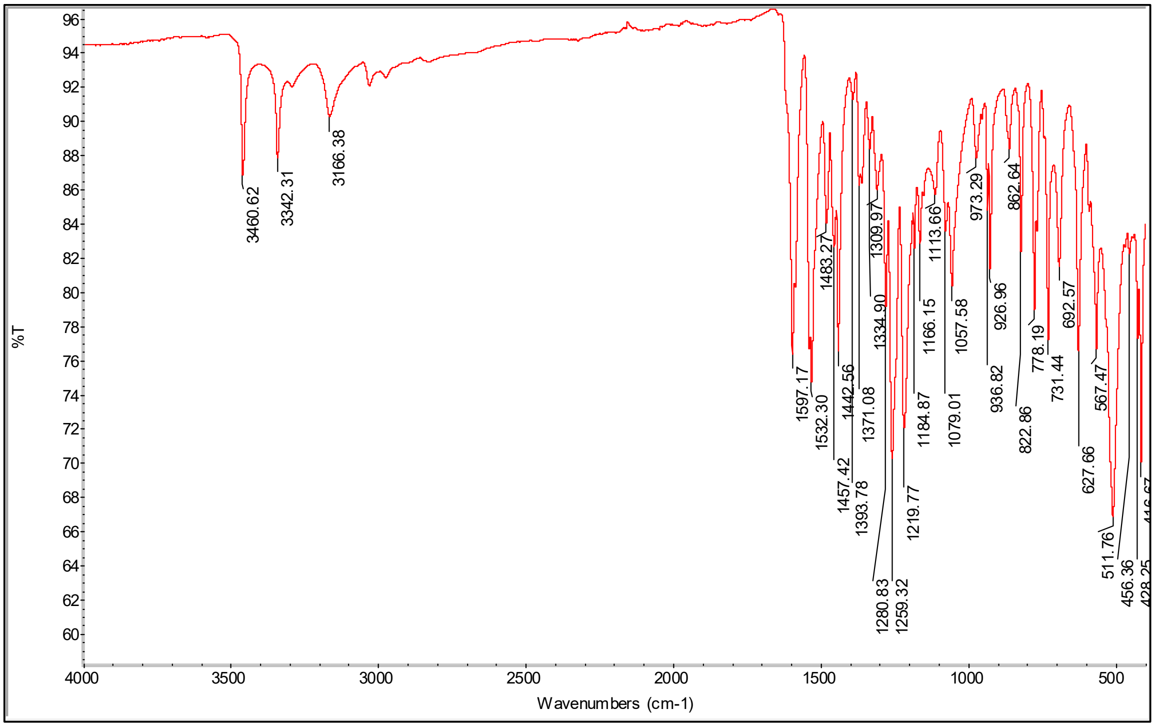

Figure S6. IR spectrum of complex **3**.

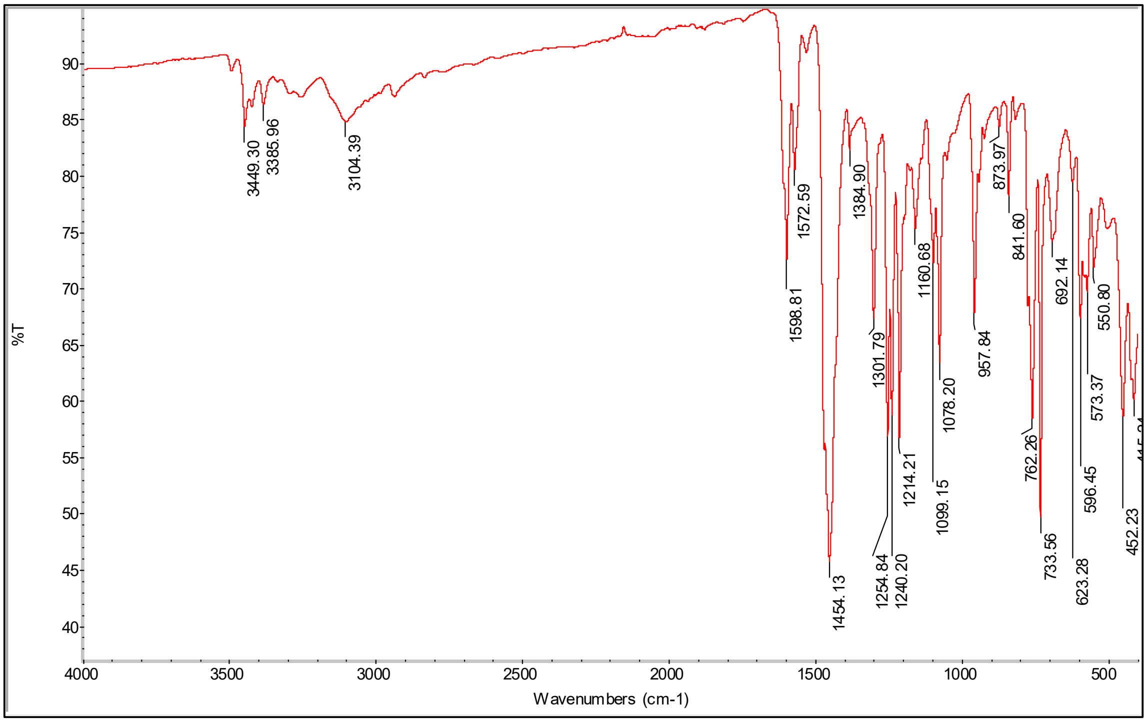

Figure S7. IR spectrum of complex **6**.

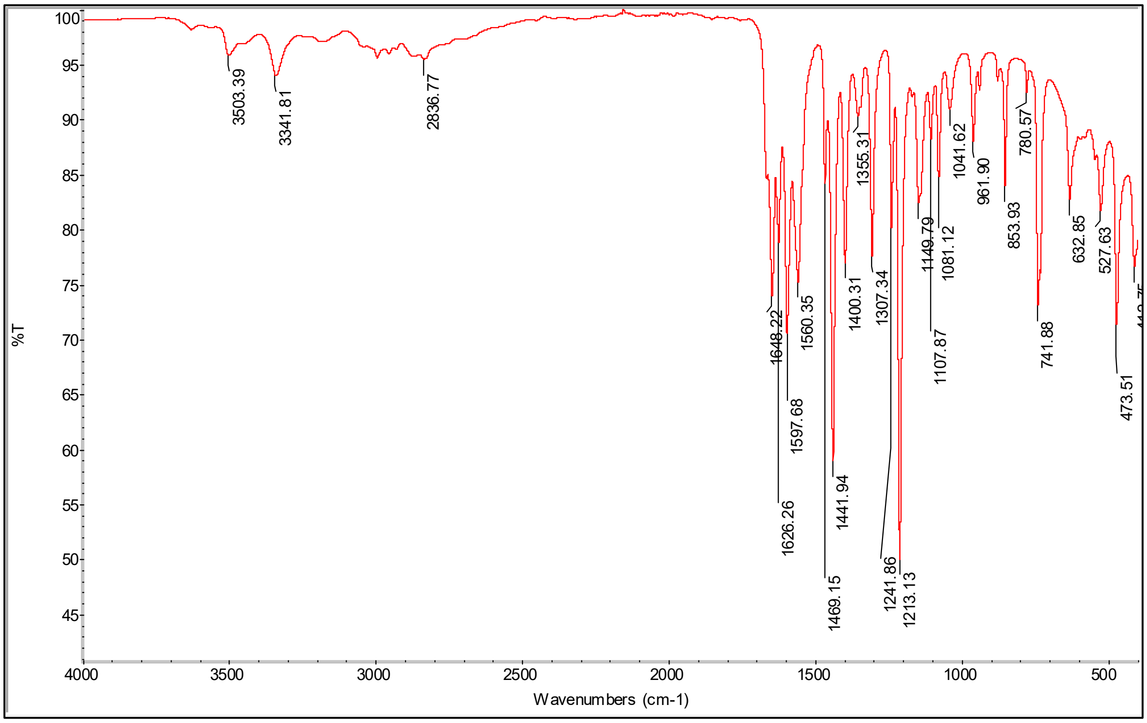

Figure S8. IR spectrum of complex **7**.

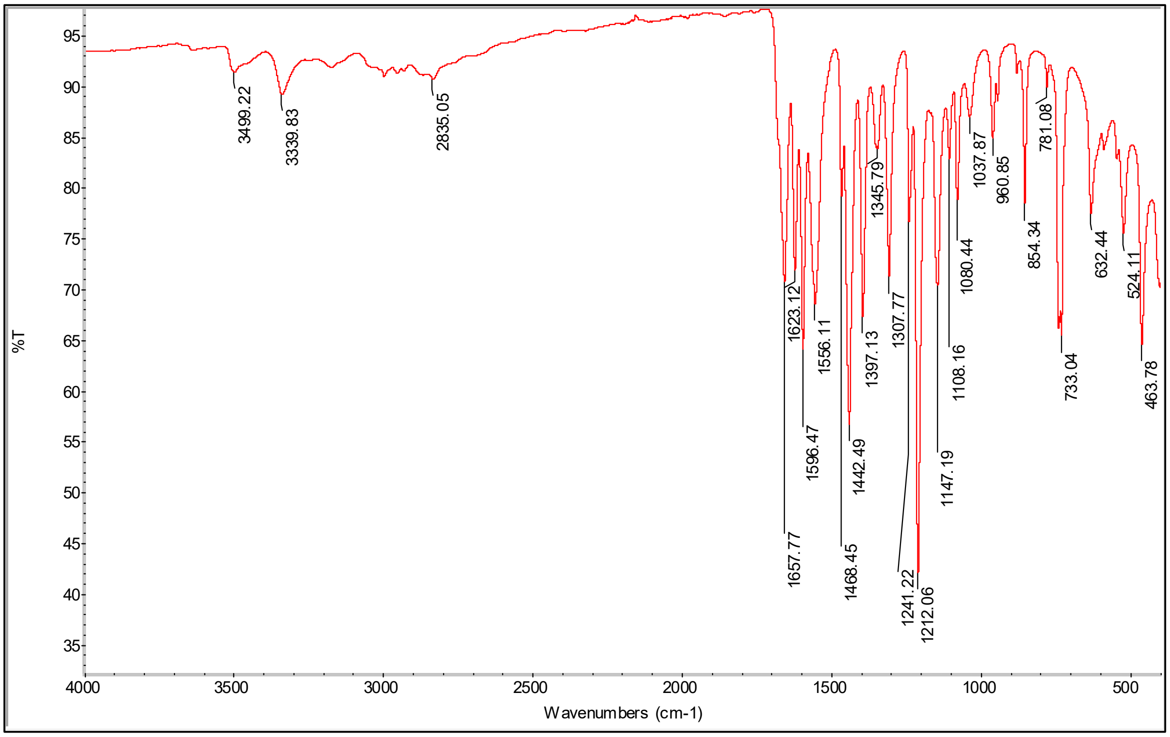

Figure S9. ^{1}H NMR (top) and ^{13}C NMR (bottom) spectrum of complex **2** registered in DMSO-d6.

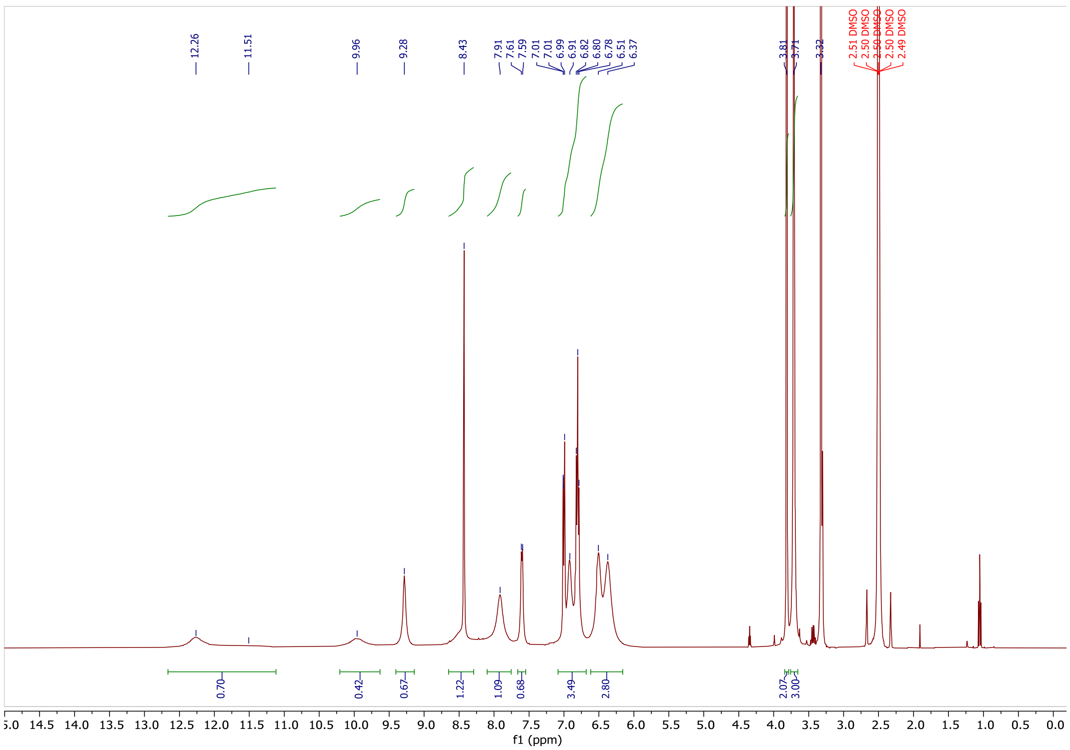

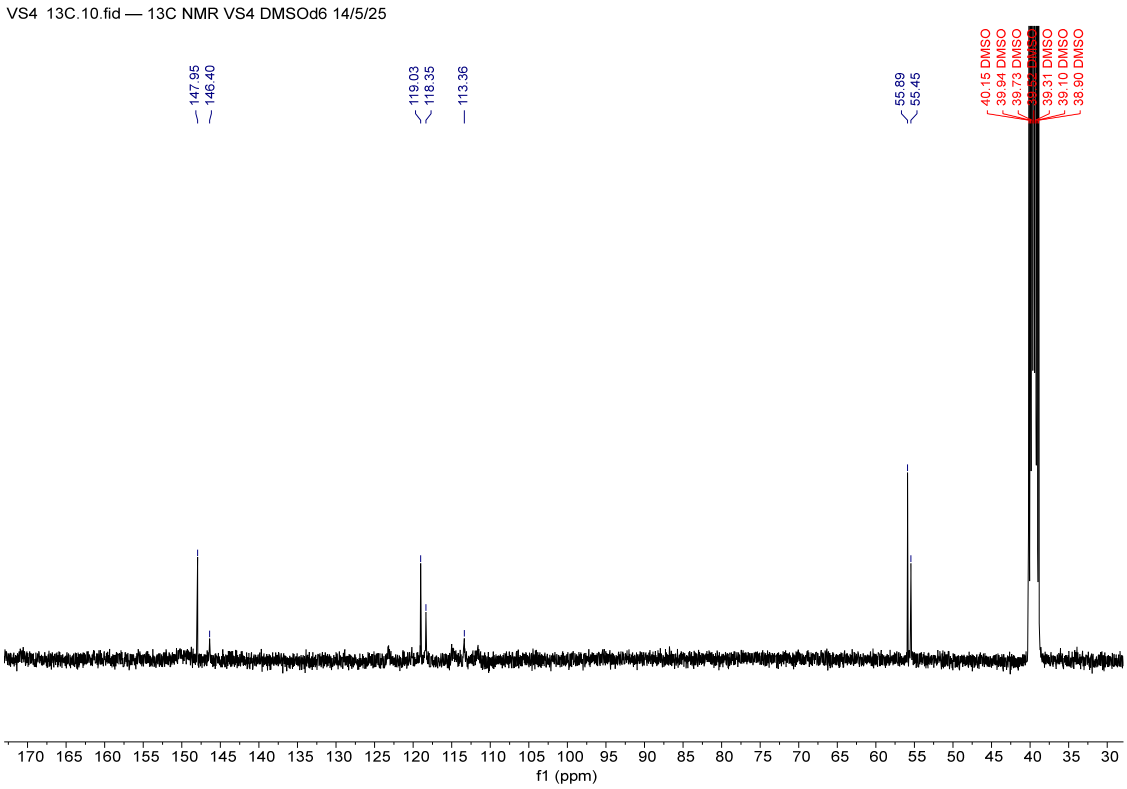

Figure S10. ^{1}H NMR spectrum of complex **7** registered in DMSO-d_6.

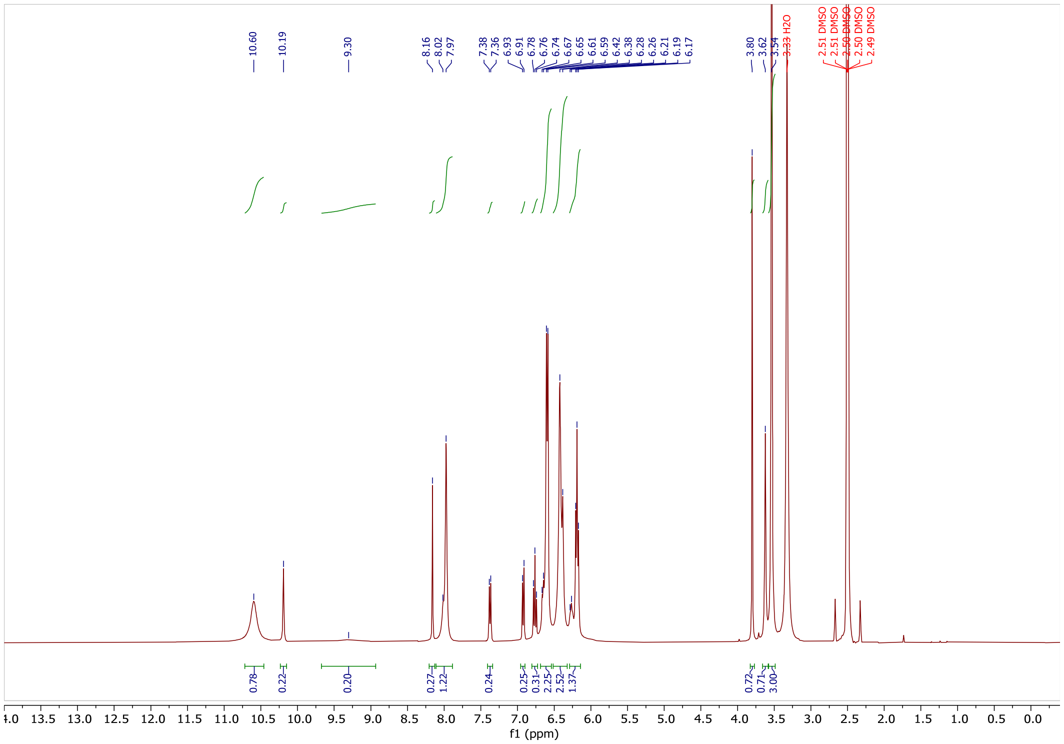

Figure S11. ^{1}H NMR spectrum of complex **12** registered in DMSO-d_6.

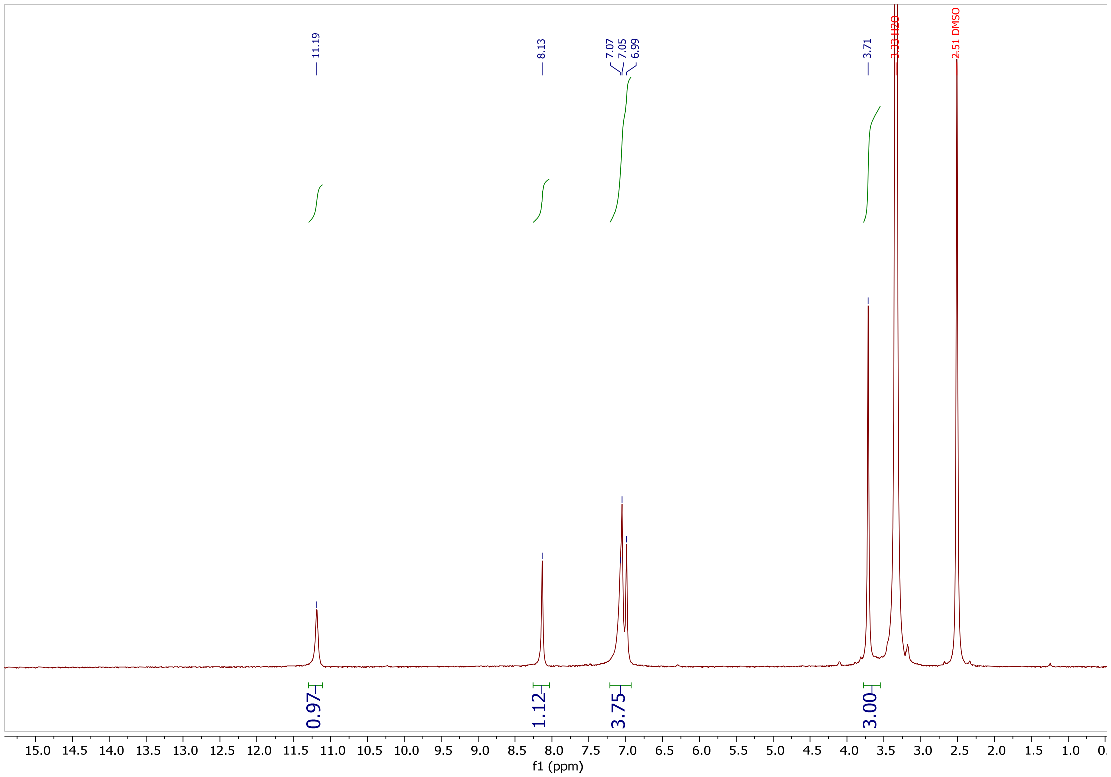

Figure S12. ^{1}H NMR spectrum of complex **14** registered in DMSO-d$_6$.

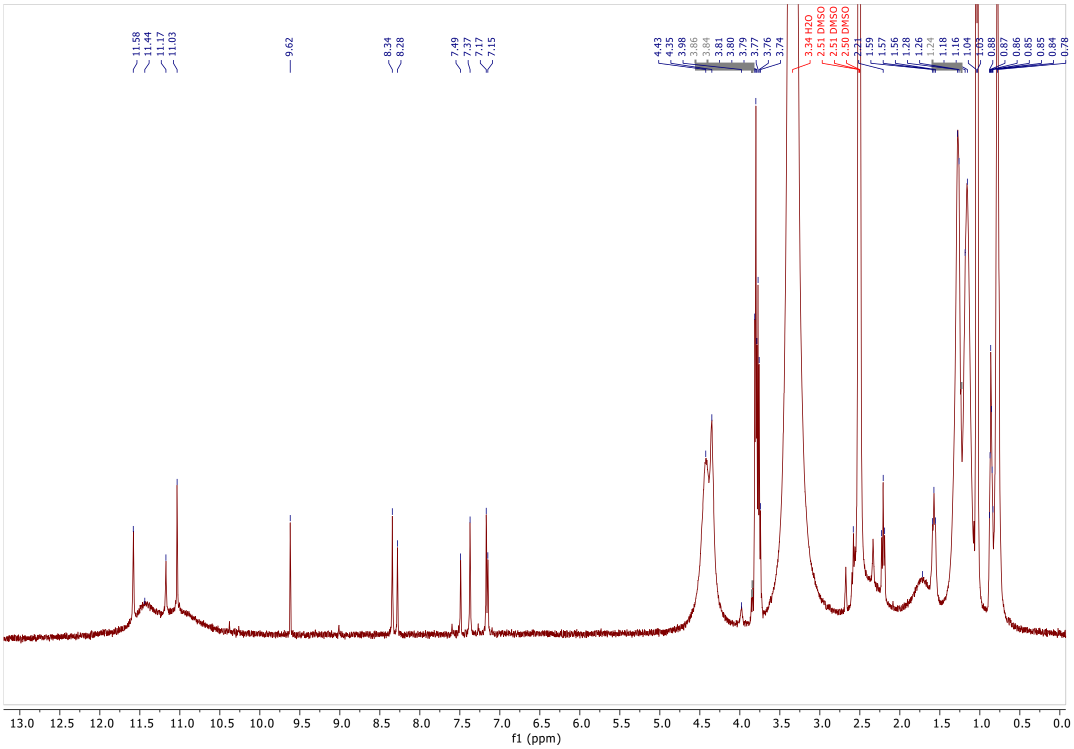

Figure S13. IR spectrum of complex **10**.

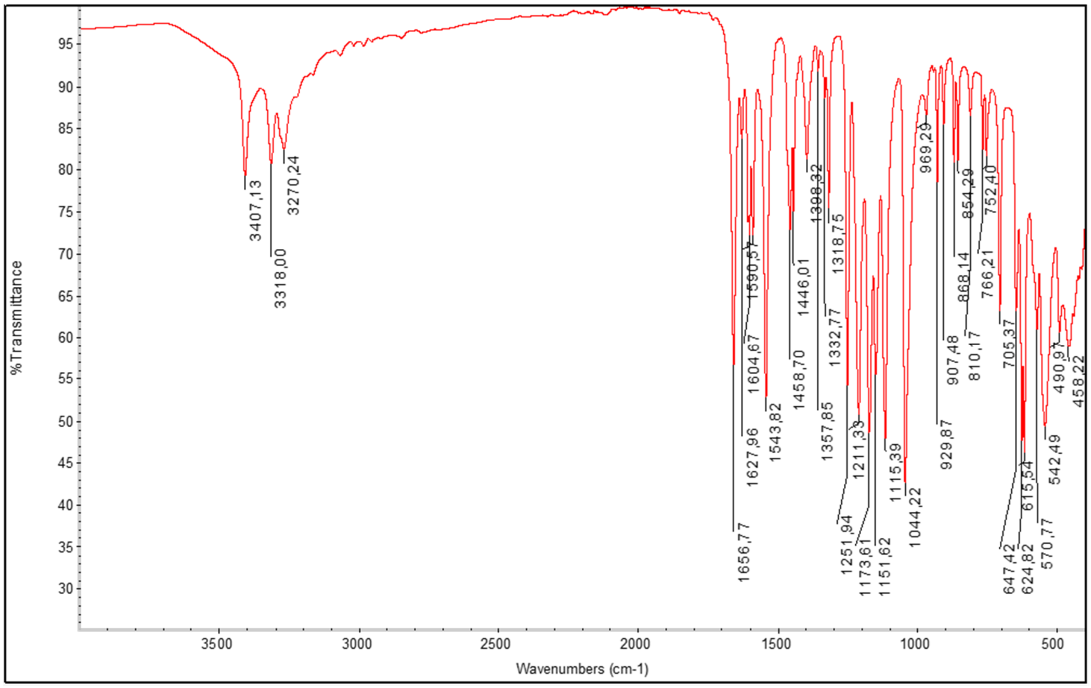

Figure S14. IR spectrum of complex **11**.

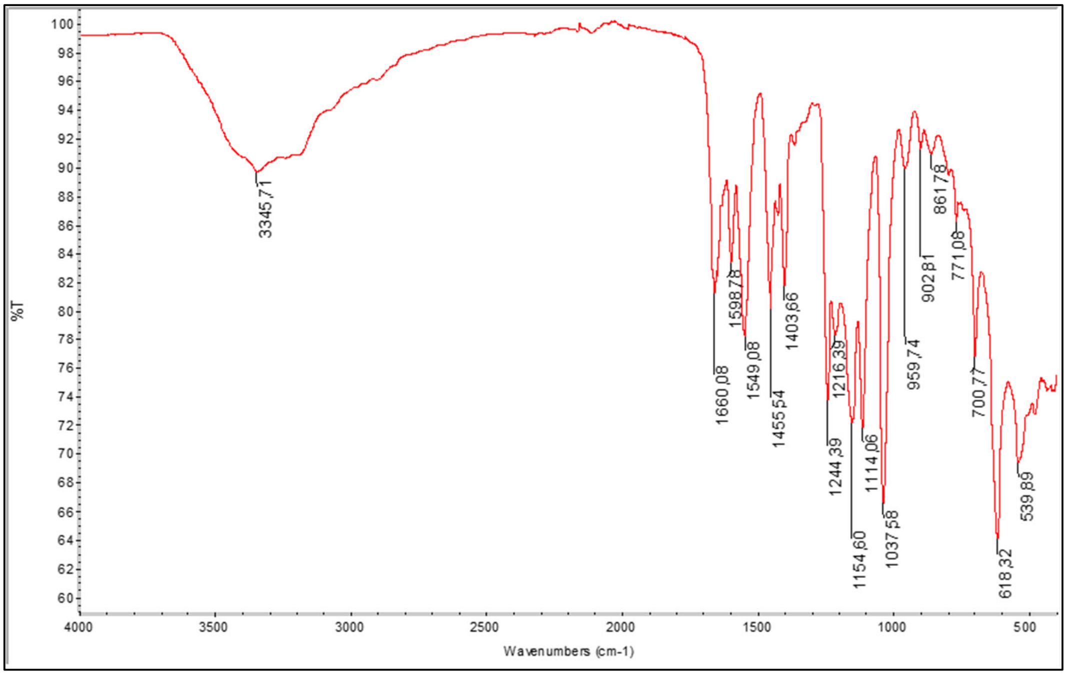

Figure S15. IR spectrum of complex **12**.

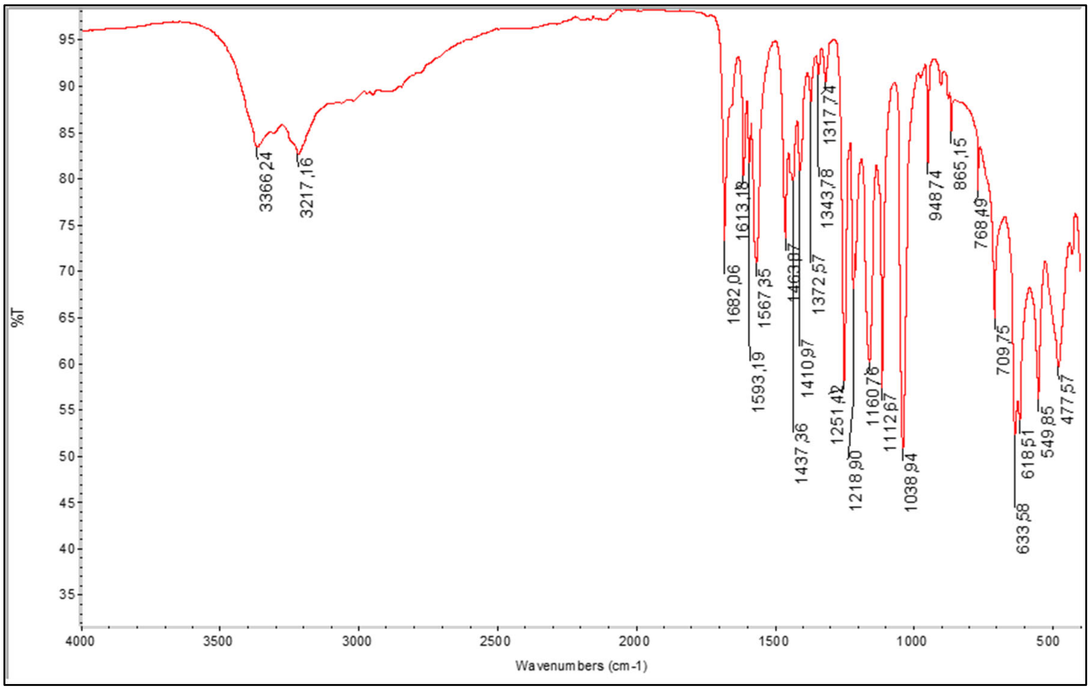

Figure S16. IR spectrum of complex **13**.

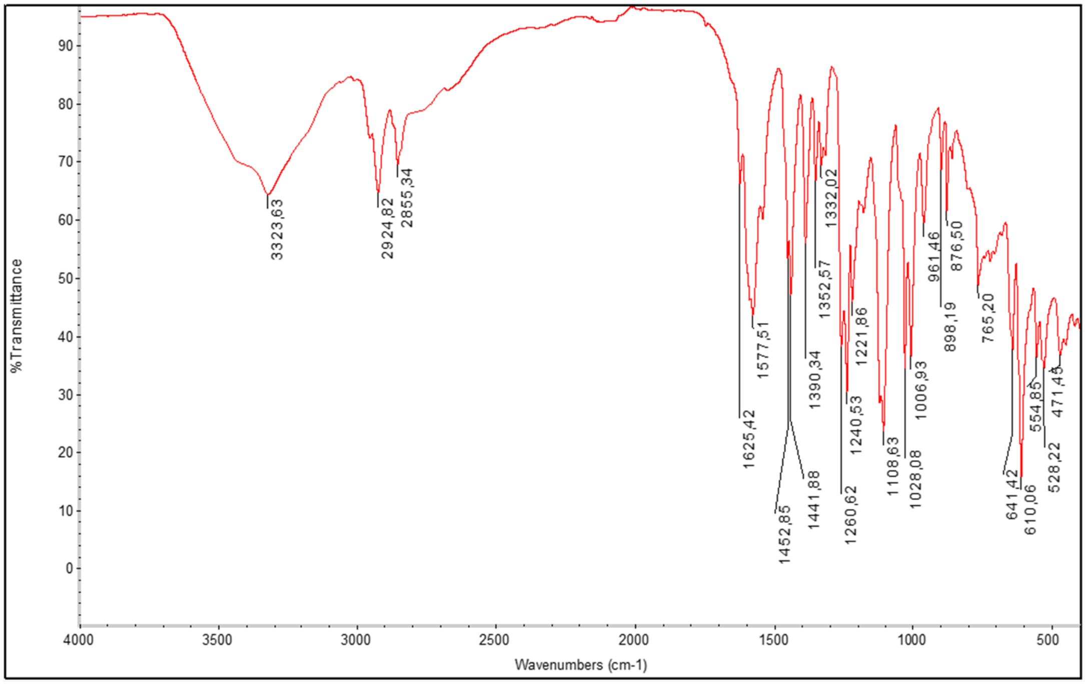

Figure S17. IR spectrum of complex**14**.

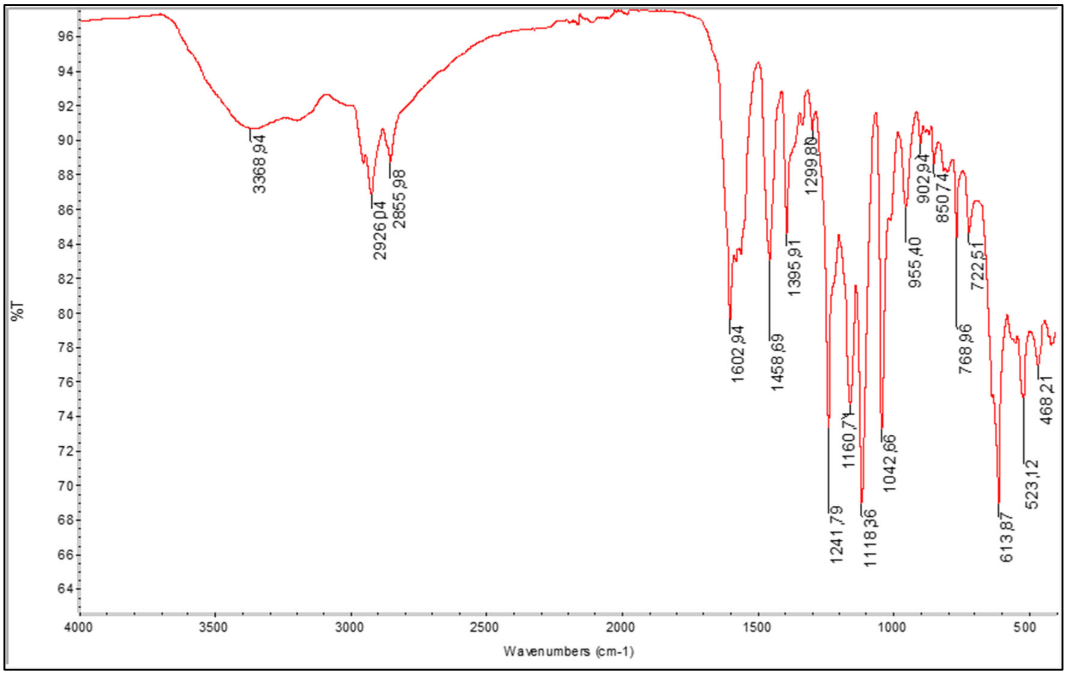

Figure S18. ESI-MS spectrum for complex **10** (negative ions; solvent: methanol).

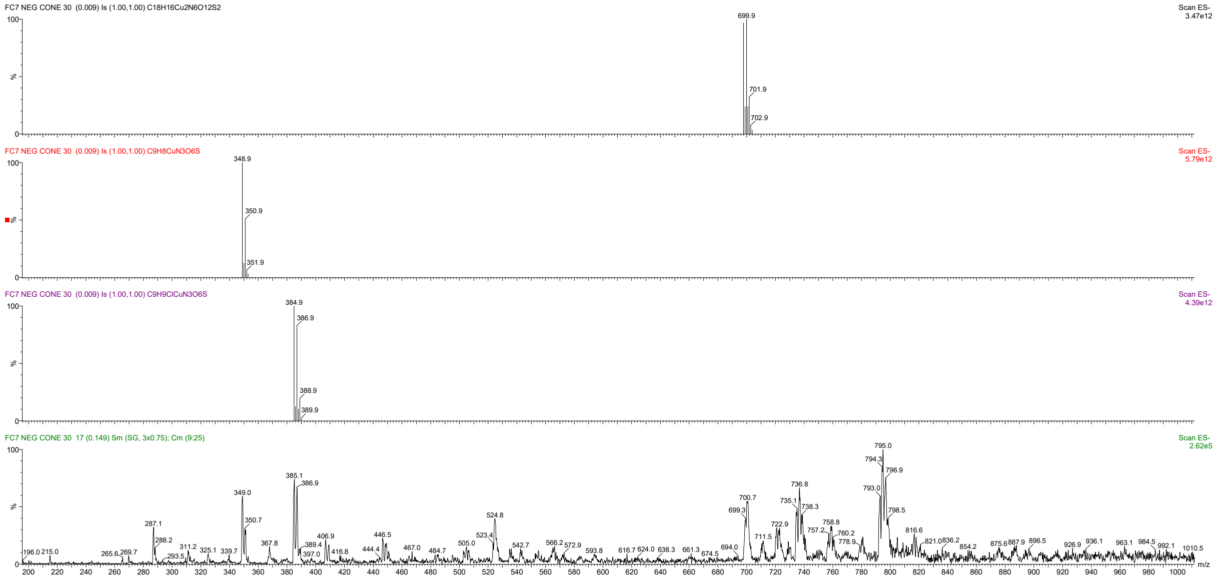

Figure S19. ESI-MS spectrum for complex **13** (negative ions; solvent: methanol).

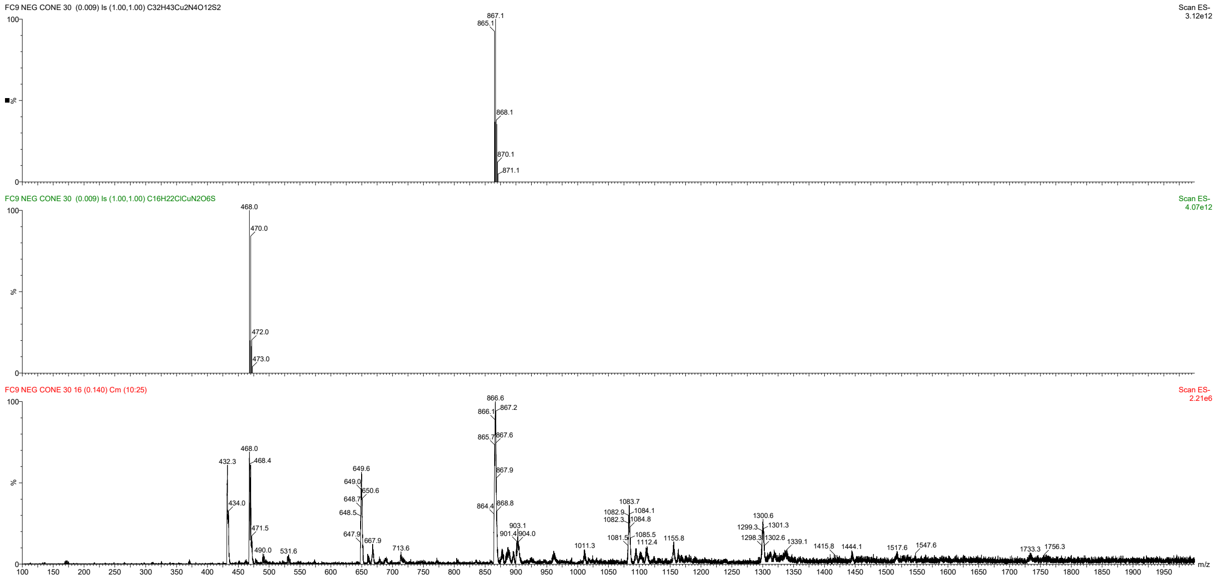

Table S1. Microbial growth and cell proliferation at 100 μM (mean values and standard deviations)

Ligand	*S. aureus*	*E. coli*	*C. albicans*	HEL 299
$\mathbf{H_2L_1}$	0.3 ± 0.5	88.9 ± 8.3	43.6 ± 6.1	94.5 ± 7.9
$\mathbf{H_2L_2}$	96.8 ± 3.5	92.8 ± 3.1	83.3 ± 14.4	97.2 ± 13.2
$\mathbf{NaH_2L_4}$	99.0 ± 3.5	100.2 ± 1.0	95.6 ± 3.2	85.6 ± 10.2
$\mathbf{NaH_2L_5}$	98.4 ± 3.0	107.4 ± 0.9	95.6 ± 7.5	90.0 ± 4.9
$\mathbf{NaH_2L_6}$	86.5 ± 1.9	97.1 ± 0.7	85.3 ± 1.6	90.3 ± 0.8
$\mathbf{NaH_2L_7}$	102.2 ± 1.9	101.9 ± 1.3	83.2 ± 2.7	87.6 ± 4.9
CIP[1]	0.7 ± 0.6	0.2 ± 0.3	n.d.[2]	n.d.
FCZ[3]	n.d.	n.d.	10.0 ± 0.6	n.d.
DMSO[4]	100.0 ± 2.5	100.0 ± 4.4	100 ± 1.5	98.5 ± 3.1
CP[5]	n.d.	n.d.	n.d.	9.2 ± 3.8

[1] CIP, ciprofloxacin at 0.6 μM; [2] n.d., not determined; [3] FCZ, fluconazole at 1.6 μM; [4] DMSO, dimethyl sulfoxide at 0.5%; [5] CP, cisplatin at 166.6 μM.

Figure S20: Cisplatin binding interaction with plasmid DNA.

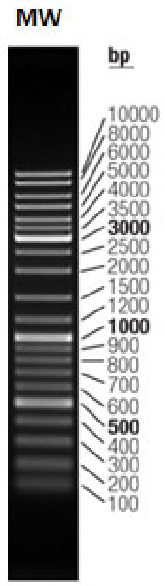

(**a**)

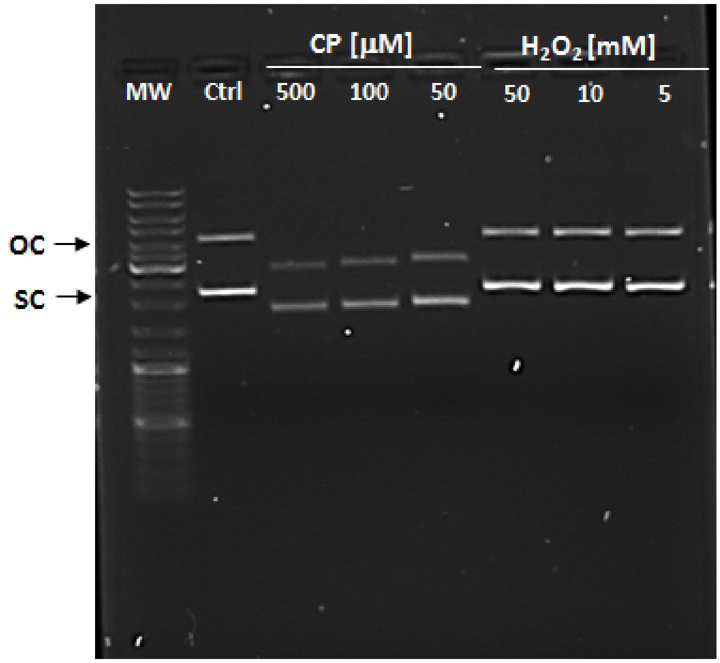

(**b**)

(a) MW, Gene Ruler DNA ladder mix (Thermo Scientific™) in a 1% agarose gel. (b) Electrophoresis analysis of pmaxGFP (Ctrl, 3527 bp) after incubation at 37°C for 2 h with cisplatin (CP) at 500 µM, 100 µM, and 50 µM, and H_2O_2 at 50 mM, 10 mM, 5 mM. Cisplatin binds covalently to the plasmid DNA forming intra-strand crosslinks that cause a shift of the electrophoresis mobility of the open circular form (OC) and the supercoiled form (SC) of the DNA. H_2O_2 does not affect DNA mobility.

Table S2. X-ray crystallographic data for compound [Ni(HL6)(H_2O)$_2$]$_2$[Ni(HL6)(H_2O)$_3$]

**Empirical formula**	C18 H30 N6 Ni2 O18 S2
**Formula weight**	800.02
**Temperature/K**	200.0
**Diffractometer/detector**	Bruker D8 Venture / PhotonII area detector
**Radiation**	CuKα (λ = 1.54178)
**Crystal system**	Monoclinic
**Space group**	$P2_1/c$
**a/Å**	9.9485(2)
**b/Å**	21.3070(5)
**c/Å**	14.1244(3)
**α/°**	90
**β/°**	96.2020(10)
**γ/°**	90
**Volume/Å^3**	2976.46(11)
**Z**	4
**ρcalc /g·cm^{-3}**	1.785
**μ/mm^{-1}**	3.710
**F(000)**	1648
**Θ range for data collection/°**	3.77 – 72.56
	-12<h<12
**Index ranges**	-26<k<26
	-15<l<17
**Reflections collected**	28031
**Unique reflections**	5828
**Parameters**	425
**Goodness-of-fit on F^2**	1.089
**Final R indexes [I>=2σ (I)]**	R=0.046 wR2=0.131
**CCDC Number**	2390359

Figure S21 ORTEP representation of [Ni(HL6)(H_2O)$_2$]$_2$[Ni(HL6)(H_2O)$_3$].

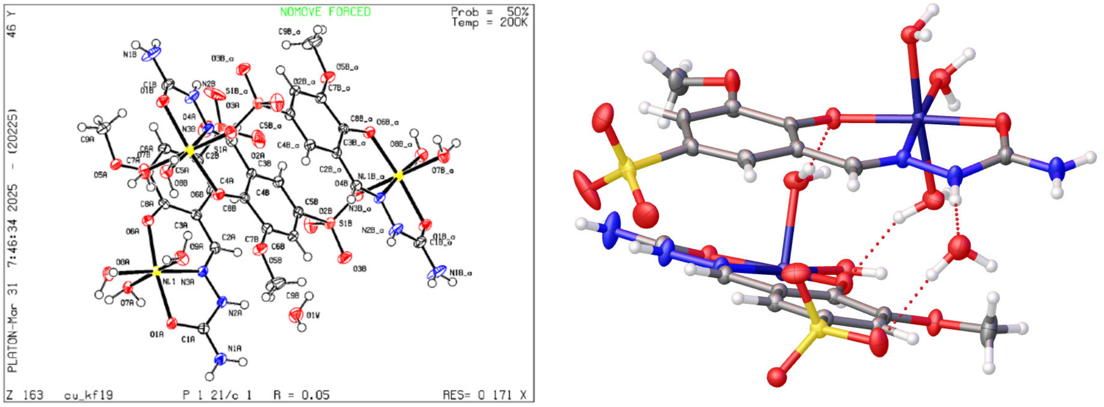

Table S3. X-ray crystallographic data for compound 12

**Empirical formula**	C9 H15 N3 O9 S Zn
**Formula weight**	406.67
**Temperature/K**	200.0
**Diffractometer/detector**	Bruker D8 Venture / PhotonII area detector
**Radiation**	CuKα (λ = 1.54178)
**Crystal system**	Monoclinic
**Space group**	$P2_1/c$
**a/Å**	9.4652(2)
**b/Å**	22.0753(4)
**c/Å**	7.1489(1)
**α/°**	90
**β/°**	99.769(1)
**γ/°**	90
**Volume/Å^3**	1472.08(5)
**Z**	4
**ρcalc /g·cm^{-3}**	1.835
**μ/mm^{-1}**	4.167
**F(000)**	832
**Θ range for data collection/°**	4.00 – 70.01
	-11<h<9
**Index ranges**	-21<k<26
	-8<l<8
**Reflections collected**	8423
**Unique reflections**	2718
**Parameters**	268
**Goodness-of-fit on F^2**	1.044
**Final R indexes [I>=2σ (I)]**	R=0.030, wR2=0.076
**CCDC Number**	2435709

Figure S22 ORTEP representation of [Zn(HL6)(H_2O)$_2$].

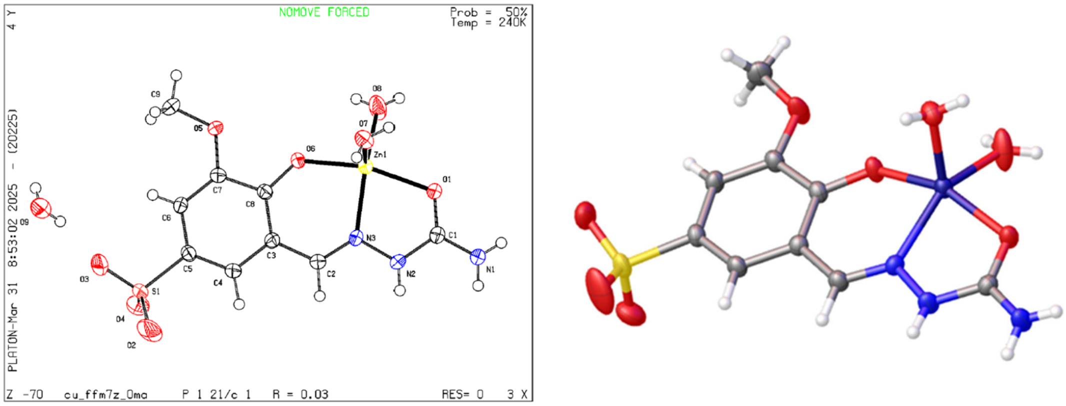

Table S4. X-ray crystallographic data for compound [Cu(HL7)(H_2O)]

**Empirical formula**	C16 H24 Cu N2 O7 S1
**Formula weight**	451.97
**Temperature/K**	200.0
**Diffractometer/detector**	Bruker D8 Venture / PhotonII area detector
**Radiation**	CuKα (λ = 1.54178)
**Crystal system**	Triclinic
**Space group**	P-1
**a/Å**	7.4069(4)
**b/Å**	8.8843(4)
**c/Å**	15.9868(8)
**α/°**	101.846(2)
**β/°**	94.907(2)
**γ/°**	110.0360(10)
**Volume/Å^3**	953.29(8)
**Z**	2
**ρcalc /g·cm^{-3}**	1.575
**μ/mm^{-1}**	3.014
**F(000)**	470
**Θ range for data collection/°**	2.87– 72.32
	-9<h<9
**Index ranges**	-9<k<10
	-19<l<19
**Reflections collected**	18750
**Unique reflections**	3753
**Parameters**	247
**Goodness-of-fit on F^2**	1.075
**Final R indexes [I>=2σ (I)]**	R=0.032 wR2=0.087
**CCDC Number**	2435710

Figure S23 ORTEP representation of the head-to-tail dimers [Cu(HL7)(H_2O)]$_2$ (left) and of the molecular unit [Cu(HL7)(H_2O)]

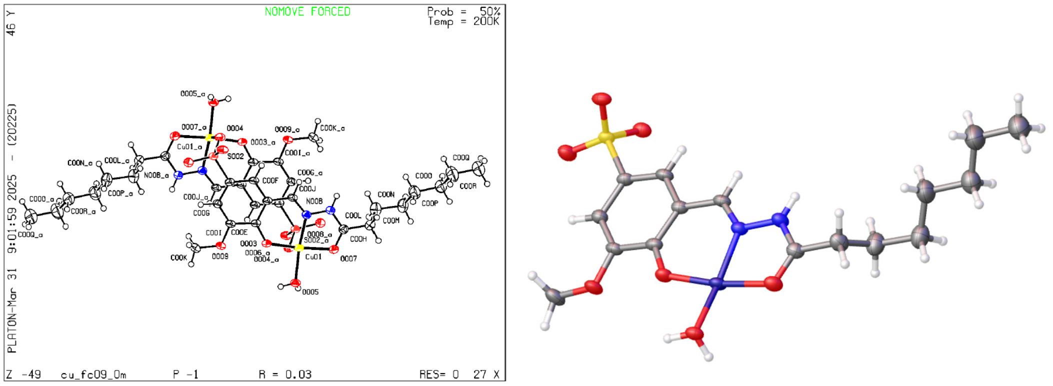

Figure S24. UV-visible spectra for compound **13** (0.25 μM) dissolved in a 25 mM HEPES/NaCl (0.9%) solution at pH 7.4 over 72 hours.

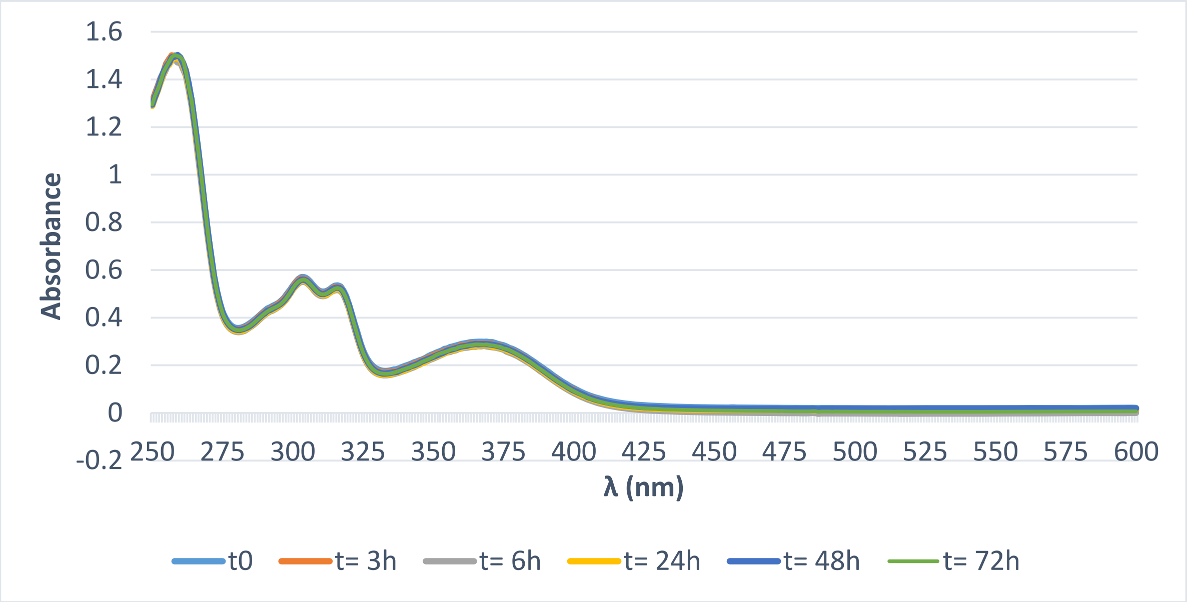

Supplement: Supplementary file 1 [file molecules-30-02329-s001.zip › molecules-3608737-supplementary.pdf]
